# Harnessing deep learning to forecast local microclimate using global climate data

**DOI:** 10.1038/s41598-023-48028-1

**Published:** 2023-11-29

**Authors:** Marco Zanchi, Stefano Zapperi, Caterina A. M. La Porta

**Affiliations:** 1 Department of Environmental Science and Policy, University of Milan, Via Celoria 10, 20133 Milano, Italy; 2 Center for Complexity and Biosystems, University of Milan, Via Celoria 16, 20133 Milano, Italy; 3 Department of Physics, University of Milan, Via Celoria 16, 20133 Milano, Italy; 4 CNR - Consiglio Nazionale delle Ricerche, Istituto di Chimica della Materia Condensata e di Tecnologie per l’Energia, Via R. Cozzi 53, 20125 Milano, Italy; 5 CNR - Consiglio Nazionale delle Ricerche, Istituto di Biofisica, Via Celoria 10, 20133 Milano, Italy; 6 Innovation For Well-Being and Environment (CRC-I-WE), University of Milan, Via Celoria 10, 20133 Milano, Italy

**Keywords:** Climate sciences, Computational science

## Abstract

Microclimate is a complex non-linear phenomenon influenced by both global and local processes. Its understanding holds a pivotal role in the management of natural resources and the optimization of agricultural procedures. This phenomenon can be effectively monitored in local areas by employing models that integrate physical laws and data-driven algorithms relying on climate data and terrain conformation. Climate data can be acquired from nearby meteorological stations when available, but in their absence, global climate datasets describing 10 km-scale areas are often utilized. The present research introduces an innovative microclimate model that combines physical laws and deep learning to reproduce temperature and relative humidity variations at the meter-scale within a study area located in the Lombardian foothills. The model is exploited to perform a comparative study investigating whether employing the global climate dataset ERA5 as input reduces model’s accuracy in reproducing the microclimate variations compared to using data collected by the Lombardy Regional Environment Protection Agency (ARPA) from a nearby meteorological station. The comparative analysis shows that using local meteorological data as inputs provides more accurate results for microclimate modeling. However, in situations where local data is not available, the use of global climate data remains a viable and reliable approach.

## Introduction

Microclimate is the ensemble of climatic conditions, as temperature and humidity, which vary over spatial scales of the order of meters^1^. This is an extremely complex non-linear phenomenon^2^ which involves an interplay between both global and local processes^3^. Notable examples of this interplay are the microclimate variations due to the solar radiation shading generated by the terrain conformation^4^, the diurnal temperature buffering caused by the presence of vegetation^2,5^ and wind sheltering effects produced by surface roughness^6^.

The study and comprehension of microclimate is of extreme importance in the context of precision agriculture^7^. Indeed, precision agriculture aims at achieving a higher level of accuracy and specificity in understanding the microclimate variations that occur within a particular area of interest. This includes considering factors such as temperature, humidity, precipitation, wind patterns, and other environmental parameters that can impact crop growth and development^8^. By accurately characterizing the microclimate, farmers can make informed decisions regarding the optimization of the irrigation strategies preventing potential water stress on crops^9^ or the optimization of windbreaks placement protecting crops from wind damage^10^. Furthermore, the precise knowledge of microclimate variations can help in the prediction and prevention of crop diseases and pests^11^. By identifying favorable conditions for pest infestation or disease outbreaks, farmers can take timely preventive measures, such as applying targeted pesticides or implementing crop rotation strategies, thus minimizing the potential damage to crops.

In the last years, precision agriculture has relied on microclimate monitoring systems based on internet-of-things (IoT) technologies^12^, which are recording systems able to collect and transmit data in real time. A detailed review of the state of the art of the application of IoT technologies in agriculture can be found in^13^. By obtaining spatially and temporally precise measurements using IoT technologies, farmers and agricultural researchers can gather data at specific points within the agricultural field^14^. To gain a broader perspective, the localized measurements collected through IoT technologies can be integrated with climate data. This integration allows for a more holistic analysis of the microclimate patterns and trends over larger regions. By leveraging physical laws, such as the principles of fluid dynamics and heat transfer, and utilizing linear data-driven methods, it becomes possible to construct detailed and continuous descriptions of the microclimate variations. Examples of these approaches can be found in^15–18^. In summary, this approach involves collecting uniform climate data, downscaling it to each point of the area of interest using physical laws, obtaining temperature measurements from local sensors, and fitting a linear regression model. The model captures the relationships between temperature variations and the energy balance, and its findings can be extrapolated to other points within the area of interest, resulting in a cohesive depiction of the temperature over the entire area of interest. These models can be useful for the simulation of future conditions^19^ or for crops management activities^20^.

Given the complexity of the microclimate, traditional physical laws that connect local variations in temperature and humidity with observable environmental factors such as radiation and wind may not accurately represent the true underlying phenomenon. The field of artificial intelligence (AI) has introduced non-linear modeling techniques, such as neural networks, that excel at unraveling complex relationships concealed within data. These advanced AI models have become valuable tools for the agricultural sector, enabling a deeper understanding of intricate non-linear connections hidden within agricultural data^21^. Neural networks, in particular, present a promising solution for obtaining a comprehensive description of temperature and humidity variations. By integrating localized sensor data with global climate information, neural networks can effectively capture the intricate relationship between these variables^22^.

The accuracy of neural networks in capturing the local physics relies heavily on the quality of the climate data used for training. This data is typically obtained from databases, such as the ERA5 database^23^, which provides values distributed in grids with 25 km-scale resolution. Unfortunately, this spatial scale may not be sufficient to accurately represent the actual climate conditions experienced in agricultural fields, which operate on a much smaller meter-scale resolution. One alternative is to rely on climate monitoring stations established by regional authorities, which may be placed near the area of interest. These stations can provide more localized and precise climate information, better reflecting the actual conditions experienced in the vicinity. However, there is a limitation when the area of interest is not situated near these monitoring stations, which hinders their effectiveness in accurately representing the climate of that specific area.

**Figure 1:**
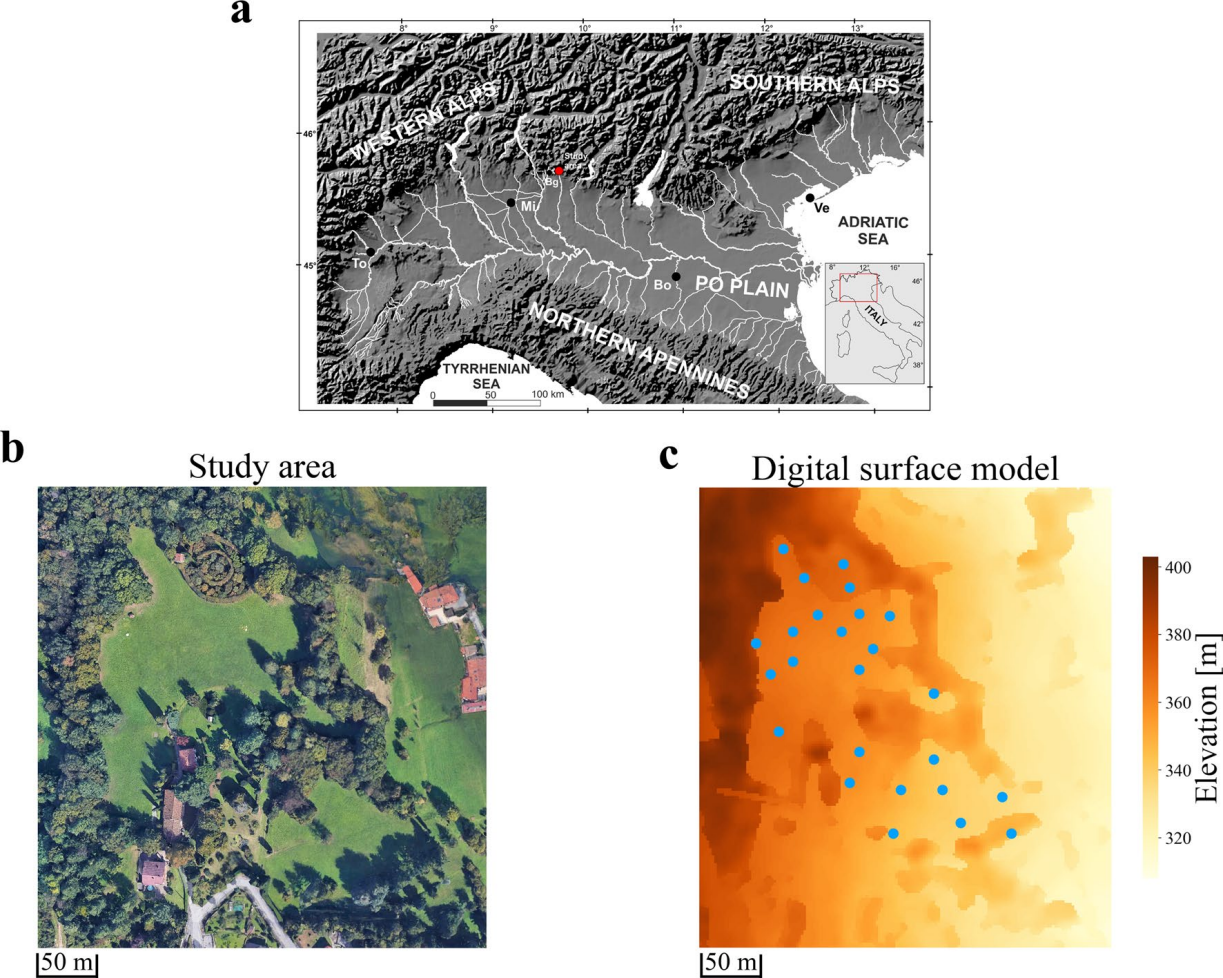
Study area. (**a**) The figure describes the location of the studied area (red point) in Northern Italy by a shaded DTM from 1:500,000 scale contours and hydrography from Touring (WA = Western Alps, EA = Eastern Alps, cSA = central Southern Alps, To = Turin, Mi = Milan, Bo = Bologna, Ve = Venice). The figure has been modified from^38^. (**b**) The figure displays the study area, which is located in the province of Bergamo in the Lombardian foothills, Italy (the center of the study area is located at $45^\circ$ 23′ 31″ N $9^\circ$ 41′ 48″ E). The image has been obtained from Google Earth Pro. (**c**) The figure describes the digital surface model at 2 m resolution of the study area (the distribution of elevation) and the location of the 25 recording sensors (blue points).

**Figure 2:**
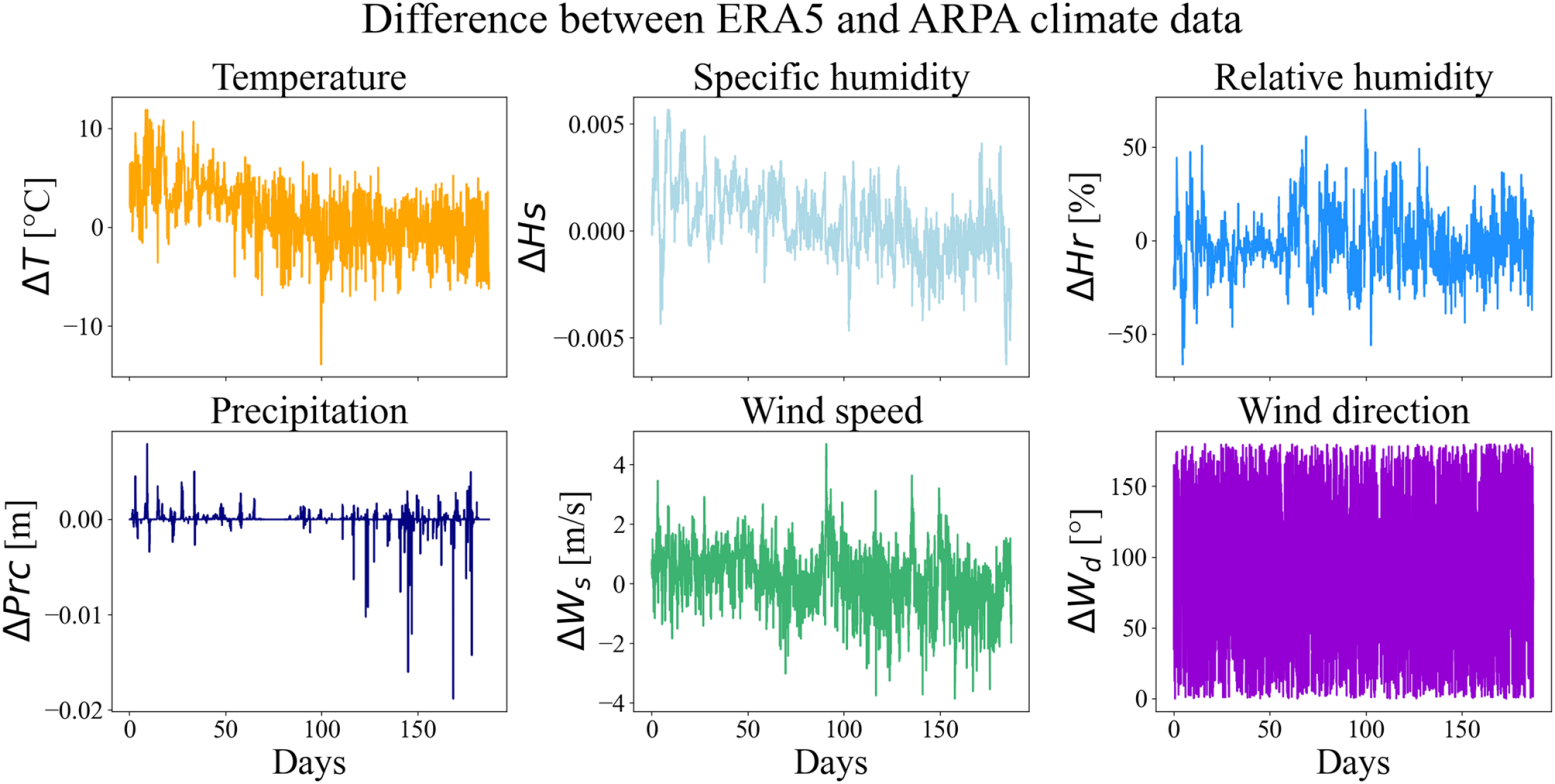
Difference between ERA5 and ARPA climate data. The figure illustrates the differences in climate data between the ERA5 database and the ARPA station recordings from November 12th, 2022, to June 22nd, 2023, with some pauses in between due to sensors maintenance (from January 15th, 2023, to February 1st, 2023, and from March 7th, 2023, to March 27th, 2023). The differences between temperature, specific humidity, relative humidity, precipitation, wind speed and wind direction are represented. The wind direction differences have been computed with a circular difference capped at $180^\circ$.

Since the literature lacks a well-established understanding of how the choice of climate data source impacts microclimate modeling, this research aims at investigating whether employing global climate data, which represents conditions over kilometer-scale areas, reduces the accuracy of microclimate modeling at the meter-scale. Specifically, this paper introduces a microclimate model which combines physical laws and neural networks to depict temperature and relative humidity variations at a meter-scale within a study area located in the province of Bergamo in the Lombardian foothills (Fig. 1). The microclimate model initially elaborates climate data with the local terrain morphology to derive meter-scale values of physical variables related to local temperature and relative humidity variations. Subsequently, feed forward neural networks have been trained, validated and tested using these variables to predict the local temperature and relative humidity over specific locations of the study area. The temperature and relative humidity data over these locations have been measured by a network of 25 sensors. The microclimate model is exploited to conduct a comparative study assessing the model’s accuracy in reproducing the local microclimate when using global climate data obtained from the 25 km-scale resolution database ERA5 versus meteorological data collected from a nearby meteorological station provided by the Lombardy Regional Environment Protection Agency (ARPA) (Fig. 2) . Four different microclimate models have been built, two for temperature prediction using data from the ERA5 database and the ARPA station, and two for relative humidity prediction. The analysis and the comparison of the models accuracy results in predicting and reproducing the local temperature and relative humidity variations reveal that for effective microclimate modeling at the meter-scale, it is preferable to utilize input climate data from a local source when available. In other cases, the use of global climate data from the ERA5 database provides a slightly less accurate but still viable option.

## Results

### Climate input modelling over the study area

**Figure 3:**
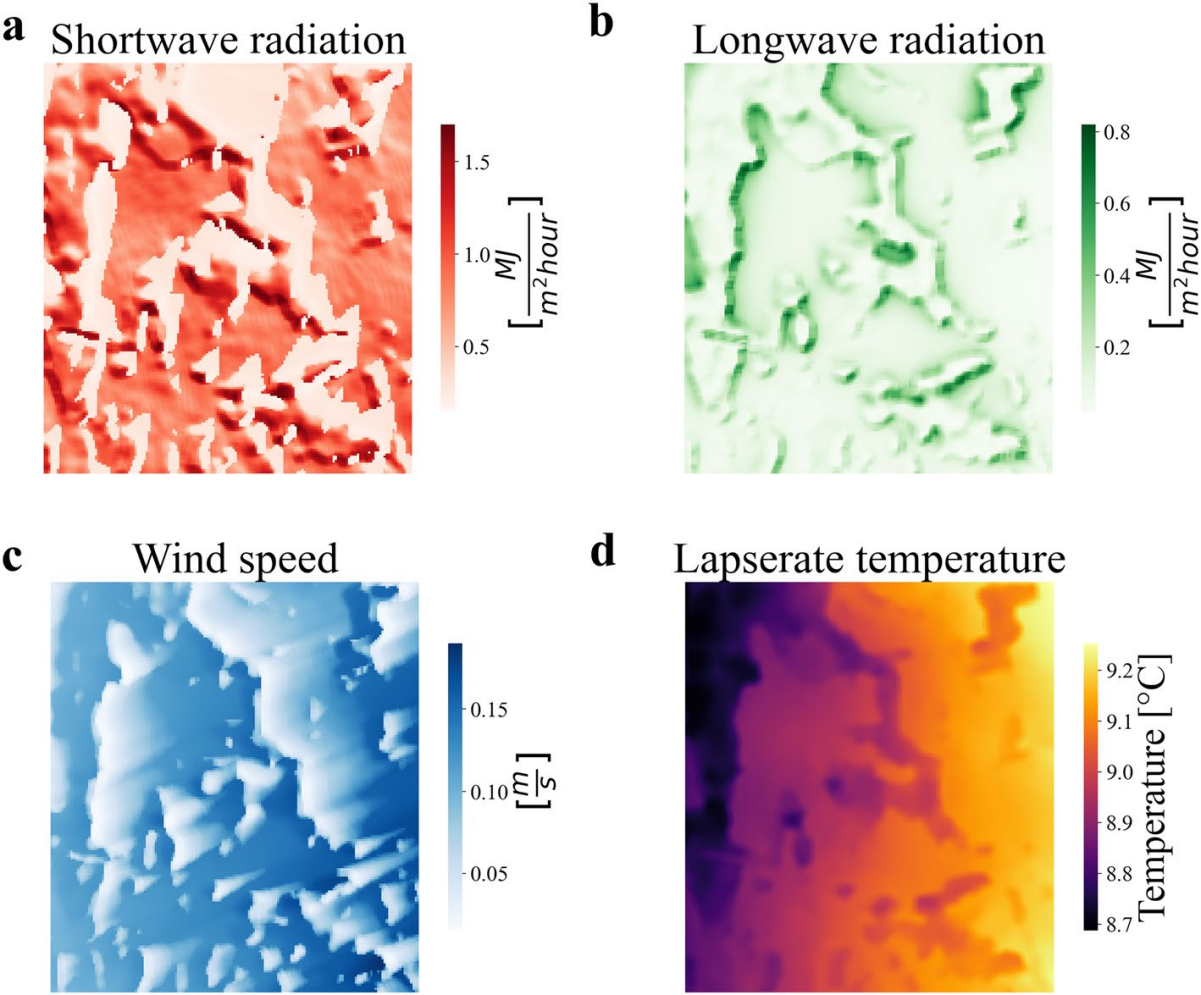
Outputs of the physical model. The following figures have been produced using the global climate data collected in the ERA5 database as inputs for the physical model. (**a**) The figure displays an example of the local variation of the short-wave radiation according to the local terrain conformation of the study area for February 2nd,2023, at 1 p.m. The variation of the short-wave radiation has been modelled as described in the methods section by Eq. (1). (**b**) The figure displays an example of the local variation of the long-wave radiation according to the local terrain conformation of the study area for February 2nd, 2023, at 1 p.m. The variation of the long-wave radiation has been modelled as described in the methods section by Eq. (9). (**c**) The figure displays an example of the local variation of the wind speed according to the local terrain conformation of the study area for February 2nd, 2023, at 1 p.m. The variation of the wind speed has been modelled as described in the methods section by Eq. (13). (**d**) The figure displays an example of the local variation of the reference temperature, corrected by the moist lapse rate according to the local terrain conformation of the study area, for February 2nd, 2023, at 1 p.m. The moist lapse rate correction has been modelled as described in the supplementary materials.

The physical microclimate modelling methodology described in the methods section has been applied to the study area to develop the physical model, describing the high resolution variation of some physical variable strictly related to the temperature and relative humidity local variation. This involves combining global climate data obtained from the ERA5 database and the ARPA station, along with the digital surface model of the study area. Initially, the physical model has been employed to generate the spatio-temporal variations of short-wave radiation, long-wave radiation, and wind speed across the entire study area from November 12th, 2022, to June 22nd, 2023. An example illustrating the spatial distribution of these variables is depicted in Fig. 3.

The outputs from the physical model demonstrate a logical and plausible representation of the physical phenomena with respect to the study area morphology described in Fig. 1c. In Fig. 3a, the lighter red regions correspond to areas shadowed by vegetation and obstacles when the sun’s azimuth is 200 degrees (specifically, on February 2nd, 2023, at 1 p.m.). Figure 3c illustrates a decrease in wind speed in the lighter blue regions, caused by the presence of vegetation and obstacles, when the wind is coming from the west (left side of the image). Additionally, Fig. 3d effectively depicts the lapse rate correction, with temperature increasing as altitude decreases.

### Temperature predictions by feed forward neural networks

Two feed forward neural networks have been trained, validated, and tested according to the methodology outlined in the feed forward neural networks section. Their purpose is to predict local temperature based on the local values of short-wave radiation, long-wave radiation, wind speed, reference temperature, reference relative humidity, precipitation, pressure, cloud cover, and specific humidity. These input variables have been obtained from the physical model using data collected from the ERA5 database and the ARPA station (combined with the ERA5 database, as explained in the meteorological data section). In the subsequent section, the results of the two training processes are initially described separately. Following that, a comparison is conducted between the two neural networks.

**Figure 4:**
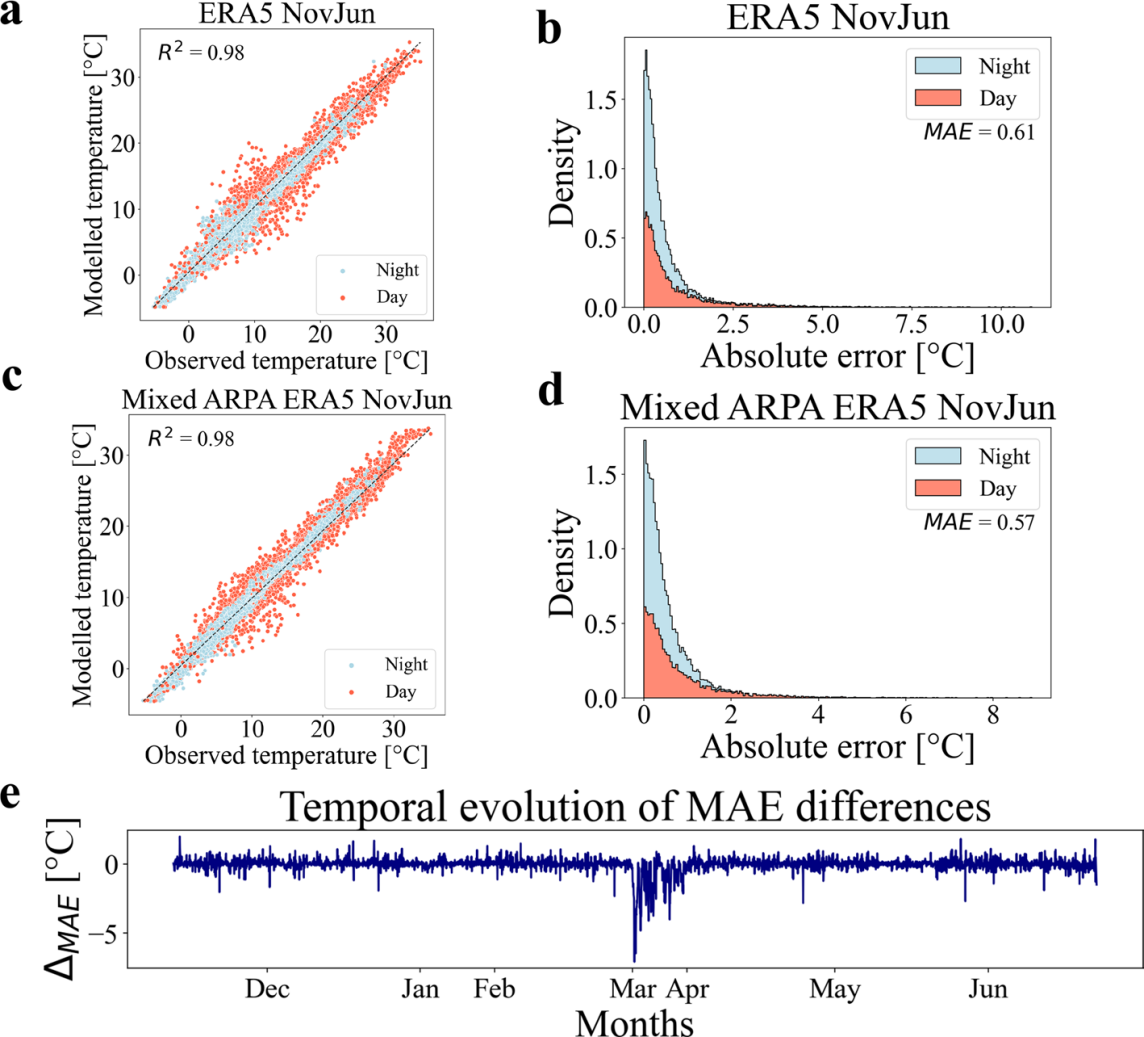
FFNN results on temperature prediction. (**a**,**c**) The figures depict the results of temperature predictions made by the feed forward neural networks on the test set using climate data from the ERA5 database and the data measured by ARPA station (combined with the ERA5 data), respectively. The Y-axis represents the neural network’s predictions, while the X-axis represents the actual temperature values. The red color represents data points during daytime, while the light blue represents data points during nighttime. (**b**,**d**) The figures illustrate the normalized distribution (scaled to have an area equal to 1) of absolute errors for temperature predictions made by the feed forward neural networks on the test set using climate data from the ERA5 database and the data measured by ARPA station (combined with the ERA5 data), respectively. The red color represents the distribution during daytime, while the light blue represents the distribution during nighttime. (**e**) The figure portrays the temporal trend of the difference between the *MAE* obtained using the data collected from the ARPA station (combined with ERA5 data) as input and the *MAE* obtained using the data stored in the ERA5 database as input. It is possible to notice that the months of January and March contain less data (these gaps correspond to the periods of sensors maintenance).

Figure 4a,b present the prediction results on the test set for the feed forward neural network trained with data collected from the ERA5 database. Figure 4a displays the temperature predictions made by the neural network on the test set, distinguishing between daytime (red color) and nighttime (light blue color) conditions. The neural network demonstrates a high level of accuracy, as indicated by the $R^2$ coefficient of 0.98. In Fig. 4b, the normalized distribution (scaled to have an area equal to 1) of absolute errors for temperature predictions is depicted. The feed forward neural network trained with ERA5 database data exhibits a very good level of prediction accuracy, with a mean absolute error (*MAE*) value of 0.61 °C. The distribution also indicates narrow tails, suggesting minimal errors for higher values. Furthermore, the performance of the neural network on the test set is compared to the predictive capability of the reference temperature. This comparison aims to evaluate whether the neural network merely replicated the reference temperature values or if it learns additional correlations within the local climate input data. By replacing the neural network’s predictions with the reference temperature values, the resulting $R^2$ value is found to be 0.79, with a *MAE* value of 2.86 °C. Comparing these values to the actual prediction results highlights that the neural network transcends the mere repetition of the reference temperature and successfully grasps some correlations among the local climate input data. To justify the use of neural networks, which are complex black-box models, their performance has been compared to that of a simple linear regression model. A linear regression has been conducted on the training set and tested on the test set, resulting in an $R^2$ value of 0.82 and a *MAE* value of 2.22 °C. These findings demonstrate that the application of a non-linear model significantly improves the accuracy of temperature predictions compared to a linear regression approach.

The prediction results on the test set for the feed forward neural network trained with data collected from the ARPA station (combined with the ERA5 database) are shown in Fig. 4c,d. In Figure 4c, the temperature predictions made by the neural network on the test set are visualized, with a distinction between daytime (red color) and nighttime (light blue color) conditions. The neural network showcases a remarkable level of accuracy, as evidenced by the $R^2$ coefficient of 0.98. Figure 4d illustrates the normalized distribution, scaled to an area equal to 1, of absolute errors for temperature predictions. The feed forward neural network demonstrates a high level of prediction accuracy with a *MAE* value of 0.57 °C. The distribution exhibits narrow tails, indicating minimal errors for higher values. The performance of the neural network has been compared to the reference temperature predictions, as illustrated previously. In this case, the resulting $R^2$ value obtained by replacing the neural network’s predictions with the reference temperature values is found to be 0.94, accompanied by a *MAE* value of 1.18 °C. As in the previous case, a linear regression has been conducted on the training set and tested on the test set, resulting in an $R^2$ value of 0.96 and a *MAE* value of 0.89 °C. In this case, the linear regression model performed reasonably well compared to the feed forward neural network. However, this could be attributed to the reference temperature having a closer value to the actual temperature, as indicated by the *MAE* value of 1.18 °C for the reference temperature prediction. This does not necessarily imply a deeper understanding of the correlations between the local temperature and the local climate variables by the linear regression model.

Now, it is possible to compare the feed forward neural network trained on climate data from the ERA5 database with the one trained on the ARPA station recordings (combined with the ERA5 database). Both neural networks have achieved a very good performance, although slightly higher prediction errors are observed during daytime time. The neural network trained on the ARPA station meteorological data exhibits slightly better accuracy, which is also reflected in the distributions shown in Fig. 4b,d, where the tails of the former network reach higher *MAE* values. Furthermore, the temporal trend of the difference between the *MAE* values of the two neural networks is depicted in Fig. 4e. A negative trend indicates that the feed forward neural network trained on the ARPA station recordings outperforms the one trained on the ERA5 database (a positive trend implies the opposite). Fluctuations around 0 are observed for most of the period, except for a brief interval with more consistent negative fluctuations occurring during March. These fluctuations have been generated by differences in the meteorological data provided by the two databases. Indeed, the ERA5 database has provided meteorological conditions indicating temperature measures around 5 °C and relative humidity values around 80$\%$, while the ARPA station and the sensors have recorded temperature values around 15 °C and relative humidity values around 40$\%$. This indicates that the ERA5 database does not accurately represent the global climate conditions measured by the nearby ARPA station. Based on these results, it appears that utilizing a feed forward neural network trained on data recorded by a climate station near the study area is preferable for replicating local temperature conditions compared to using globally uniform climate data from the ERA5 database, which has a 25 km grid resolution. However, the results obtained from training on the ERA5 database are still good. Therefore, in cases where meteorological stations are not available, local temperature predictions can also be performed using a global climate database like ERA5.

### Relative humidity predictions by feed forward neural networks

**Figure 5:**
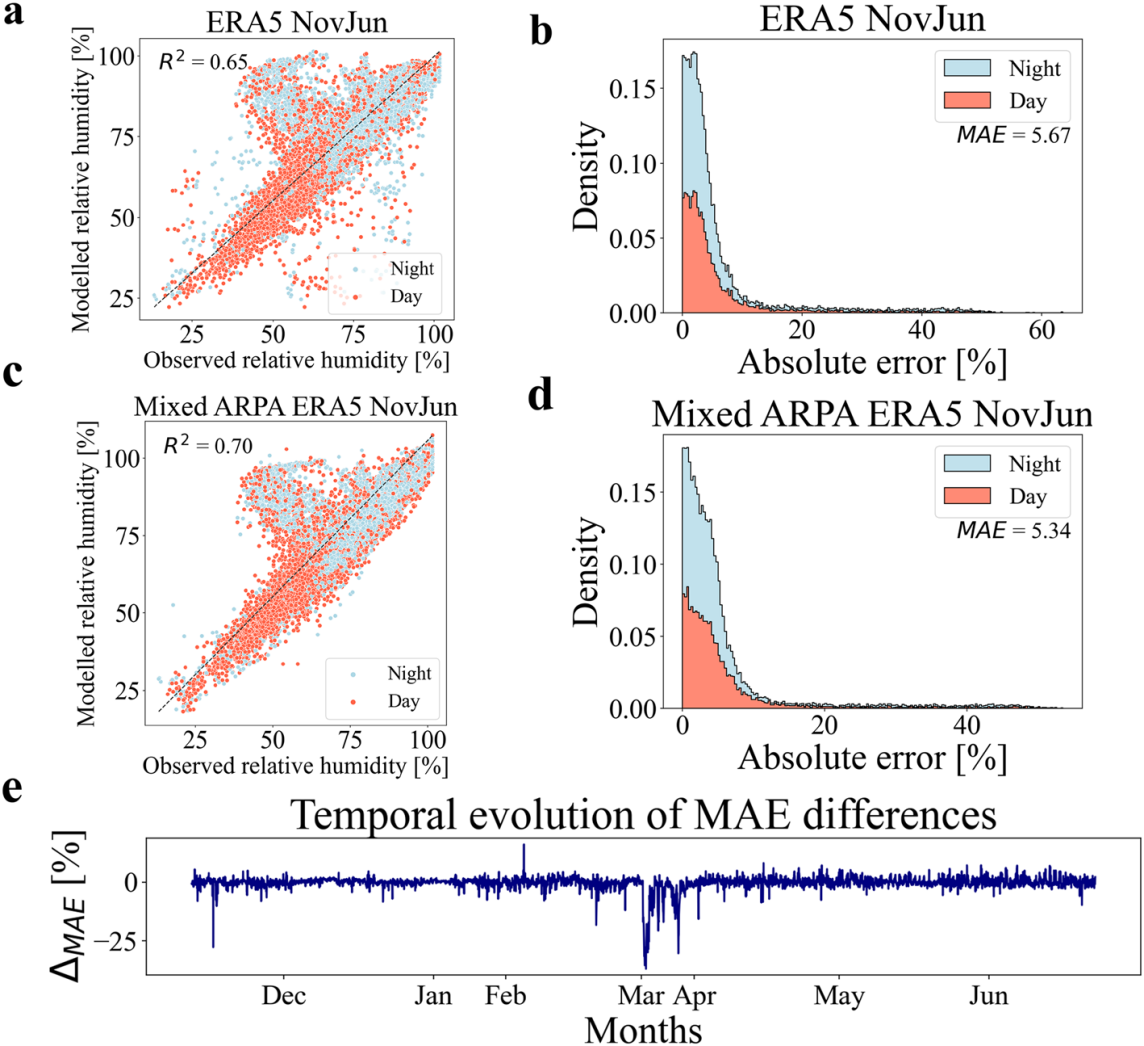
FFNN results on relative humidity prediction. (**a**,**c**) The figures depict the results of relative humidity predictions made by the feed forward neural networks on the test set using climate data from the ERA5 database and the data measured by ARPA station (combined with the ERA5 data), respectively. The Y-axis represents the neural network’s predictions, while the X-axis represents the actual relative humidity values. The red color represents data points during daytime, while the light blue represents data points during nighttime. (**b**, **d**) The figures illustrate the normalized distribution (scaled to have an area equal to 1) of absolute errors for relative humidity predictions made by the feed forward neural networks on the test set using climate data from the ERA5 database and the data measured by ARPA station (combined with the ERA5 data), respectively. The red color represents the distribution during daytime, while the light blue represents the distribution during nighttime. (**e**) The figure portrays the temporal trend of the difference between the *MAE* obtained using the data collected from the ARPA station (combined with ERA5 data) as input and the *MAE* obtained using the data stored in the ERA5 database as input. It is possible to notice that the months of January and March contain less data (these gaps correspond to the periods of sensors maintenance).

The procedure discussed in the previous section has been applied to predict humidity instead of temperature. To this end, two feed forward neural networks have been trained and tested to predict local relative humidity using local measurements using data collected from the ERA5 database and the ARPA station. Figure 5a,b showcase the outcomes of the test set predictions by the feed forward neural network trained on data from the ERA5 database. In Fig. 5a, the predicted relative humidity values are presented, distinguishing between daytime (red color) and nighttime (light blue color) conditions. The neural network demonstrates a moderate level of accuracy, with an $R^2$ coefficient of 0.65. However, there is a cluster of incorrect predictions in the upper center part of the figure. Figure 5b illustrates the normalized distribution (scaled to have an area equal to 1) of the absolute errors for relative humidity predictions. The feed forward neural network trained on ERA5 database data shows a reasonable level of prediction accuracy, with a mean absolute error (*MAE*) value of 5.67$\%$. Additionally, the performance of the neural network on the test set is compared to the predictive capability of the reference relative humidity. When replacing the neural network’s predictions with the reference relative humidity values, the resulting $R^2$ value is only 0.001, with a high *MAE* value of 63.78$\%$. These values indicate that the reference relative humidity is not a reliable representation of the actual local conditions. As seen in the previous section, a linear regression model was also applied to the training set and tested on the test set. The linear regression yielded an $R^2$ value of 0.26 and a *MAE* value of 11.13$\%$. These results further emphasize the significance of employing a non-linear model, such as the feed forward neural network, to achieve improved accuracy in predicting relative humidity compared to linear regression.

The prediction results for the feed forward neural network trained on data from the ARPA station (combined with the ERA5 database) are presented in Figure 5c,d. Figure 5c visualizes the relative humidity predictions made by the neural network on the test set, distinguishing between daytime (red color) and nighttime (light blue color) conditions. The neural network demonstrates a fairly good level of accuracy, indicated by the $R^2$ coefficient of 0.70. However, there is a cluster of incorrect predictions in the upper central part of the figure. In Fig. 5d, the normalized distribution of absolute errors for relative humidity predictions is displayed. The feed forward neural network exhibits a good level of prediction accuracy, with a mean absolute error (*MAE*) value of 5.34$\%$. Similarly to the previous case, the performance of the neural network is compared to the reference relative humidity predictions. When replacing the neural network’s predictions with the reference relative humidity values, the resulting $R^2$ value is 0.06, accompanied by a high *MAE* value of 64.92$\%$. As seen before, a linear regression model is applied to the training set and tested on the test set. The linear regression yields an $R^2$ value of 0.66 and a *MAE* value of 6.20$\%$. In this case, the linear regression model performs reasonably well compared to the feed forward neural network.

Now, it is possible to compare the feed forward neural network trained on climate data from the ERA5 database with the one trained on the ARPA station recordings (combined with the ERA5 database). Both neural networks have achieved satisfactory performance. However, notable prediction errors are present in both cases, as indicated by the clusters of incorrect predictions in the upper central regions of Fig. 5a,c. The fact that this pattern appears in both neural networks trained on different input data suggests the possibility of inaccurate sensor measurements for this cluster of wrong predictions. Regarding relative humidity, the neural network trained on the ARPA station meteorological data exhibits slightly better accuracy, which is also reflected in the distributions shown in Fig. 5b,d, where the tails of the former network reach higher *MAE* values. Additionally, the temporal trend of the difference between the *MAE* values of the two neural networks is depicted in Fig. 5e. Similar to the temperature case, fluctuations around 0 are observed for most of the period, except for a brief interval with more consistent negative fluctuations during March, 2023. These fluctuations have been generated by differences in the meteorological data provided by the two databases, as explained in the previous section. This suggests that the ERA5 database does not accurately represent the climate conditions measured by the nearby ARPA station. Based on these results, it appears that predicting local relative humidity is a more challenging task than temperature prediction, which may be attributed to the need for higher-quality sensor measurements. However, it is evident that utilizing a feed forward neural network trained on data recorded by a climate station near the study area is preferable for replicating local relative humidity conditions compared to using globally uniform climate data from the ERA5 database, which has a 25 km grid resolution. Nonetheless, the results obtained from training on the ERA5 database are still satisfactory in this case. Therefore, in situations where meteorological stations are unavailable, local relative humidity predictions can also be performed using a global climate database such as ERA5.

### Comparison between feed forward neural networks inputs importance

**Figure 6:**
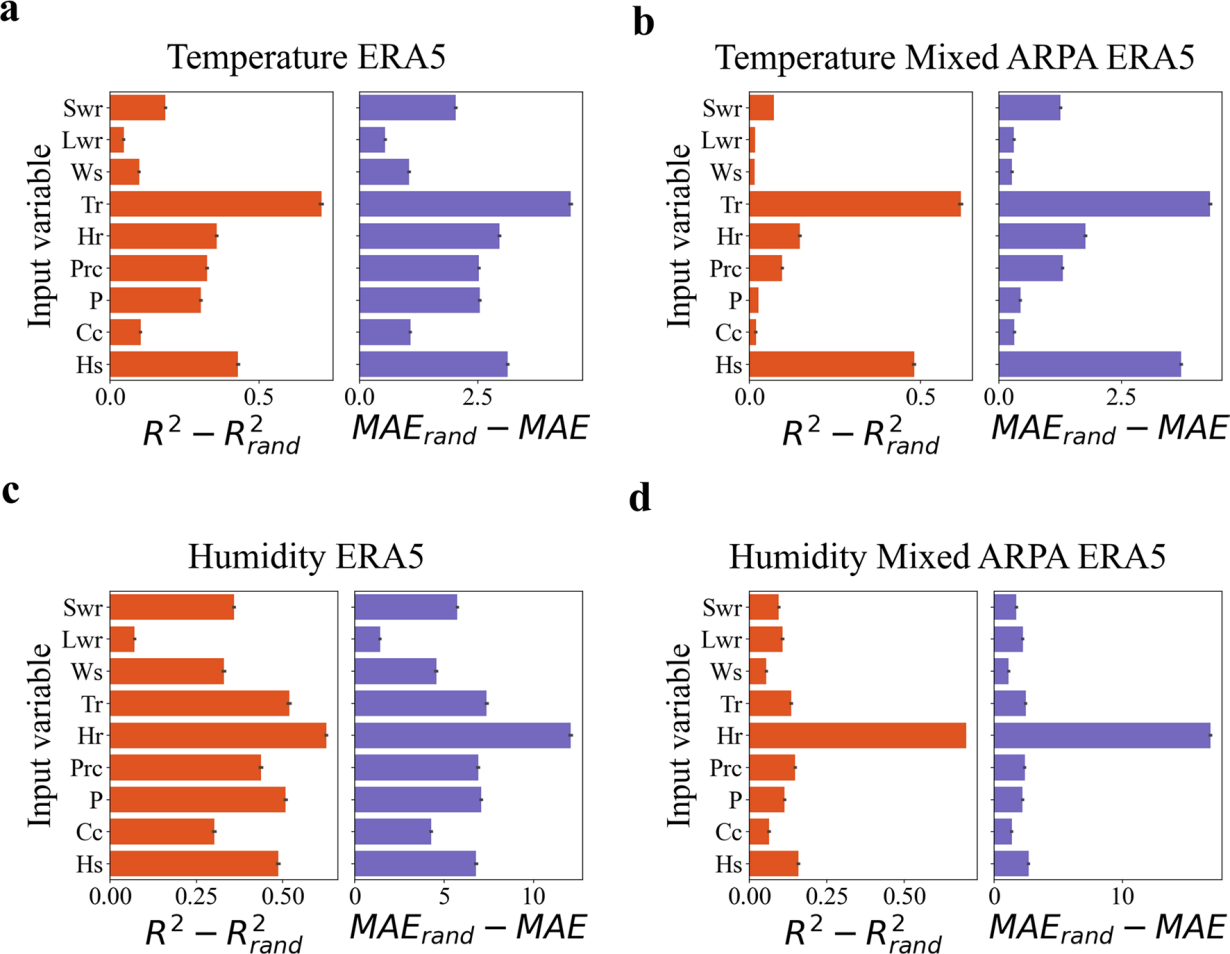
Assessment of importance of input variables. The figures provide insights into the analysis of input variable importance for the four feed forward neural networks. Each figure follows a consistent structure, with the input physical variables displayed on the Y-axis and two subplots (Swr is the shortwave radiation, Lwr is the longwave radiation, Ws is the wind speed, Tr is the reference temperature, Hr is the relative humidity, Prc is the precipitation, P is the pressure, Cc is the cloud coverm, Hs is the specific humidity). The left subplot shows the variation in $R^2$ values, while the right subplot presents the variation in *MAE* values. The bars represent the average variation across 10 simulations, and the error bars indicate the relative standard deviation of the mean. (**a**) **The figure** presents the results for the feed forward neural network trained on the ERA5 database for temperature prediction. (**b**) **The figure** showcases the results for the feed forward neural network trained on the ARPA station recordings for temperature prediction. (**c**) **The figure** displays the results for the feed forward neural network trained on the ERA5 database for relative humidity prediction. Finally, (**d**) **the figure** presents the results for the feed forward neural network trained on the ARPA station recordings for relative humidity prediction.

To address the inherent black-box nature of neural networks, a methodology for interpreting the significance of input variables on the output has been introduced in the methods section (neural networks interpretation section). This approach has been applied to the four feed forward neural networks, and the outcomes are presented in Fig. 6. For the feed forward neural networks trained on data from the ERA5 database (Fig. 6a,c), a similar pattern is observed. The importance appears to be evenly distributed among the various physical variables, with particular emphasis on the temperature and humidity reference values. Interestingly, the long-wave radiation does not seem to play a significant role in microclimate modeling. This is relevant because the modeling of longwave radiation has assumed that the difference between air and surface temperature is small (Eq. 9). While this might not be true, longwave radiation has minimal influence on neural network accuracy. Therefore, this assumption should not introduce additional errors in microclimate prediction. Similarly, the feed forward neural networks trained on the ARPA station recordings (combined with the ERA5 database) exhibit a comparable behavior (Fig. 6b,d). In both cases, the reference temperature and humidity have the highest importance, while the other variables are of secondary importance. These findings suggest that the models trained on data from the ERA5 database attempt to incorporate and correlate almost all available physical climate variables. On the other hand, the neural networks trained on the ARPA station recordings (combined with the ERA5 database) prioritize the reference parameters and likely adjust them to the local values by leveraging the other physical variables.

### Prediction over the whole study area

**Figure 7:**
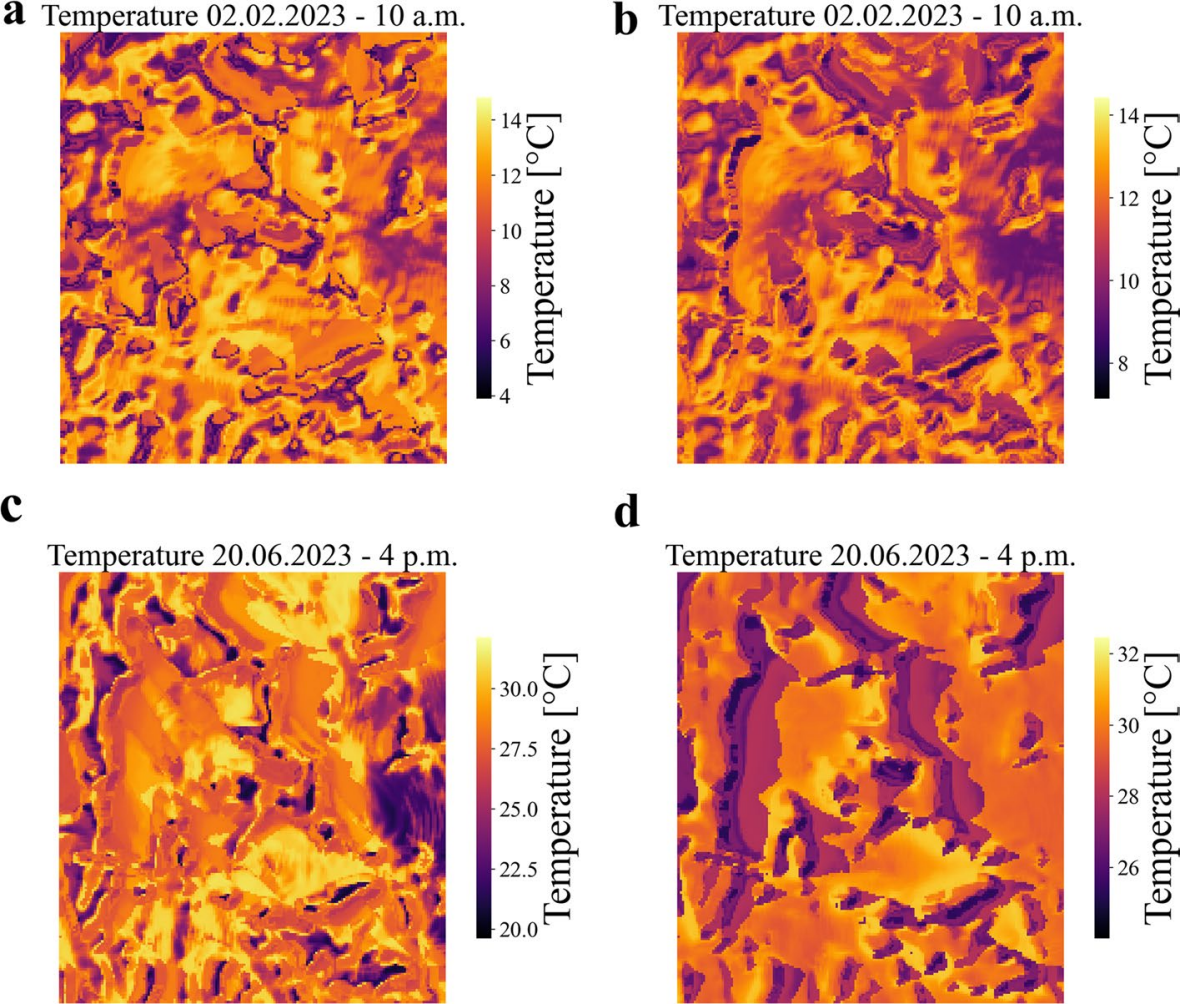
Temperature prediction over the entire study area. The figures depict the local temperature variation across the entire study area at two specific time points: February 2nd, 2023, at 10 a.m. (**a**,**b**), and June 20th, 2023, at 4 p.m. (**c**,**d**). Figures **a** and **c** show the results obtained from the feed forward neural network trained on the ERA5 database, while figures  **b** and **d** present the outcomes from the feed forward neural network trained on the data collected by the ARPA station.

After training the feed forward neural networks to predict temperature and relative humidity at the sensor location, it becomes possible to extend the predictions to cover the entire study area. Examples of temperature prediction are illustrated in Figure 7, depicting temperature maps for February 2nd, 2023, at 10 a.m. and June 20th, 2023, at 4 p.m. for both feed forward neural networks trained on the ERA5 database and the data collected from the ARPA station (combined with the ERA5 database). Given the high level of prediction accuracy achieved by both models, it can be assumed that they are capable of capturing the real temperature variations across the study area. However, considering the morphology of the study area depicted in Fig. 1, the most reliable and physically intuitive results are obtained from the feed forward neural network trained on the data collected by the ARPA station (combined with the ERA5 database). This is evident in Fig. 7b,d, where this model successfully reconstructs the regions with lower local temperatures (indicated by darker regions) caused by shadows cast by vegetation and obstacles. These shadows occur due to the sun’s azimuthal direction, with east-south direction in the morning and west in the afternoon. In contrast, the feed forward neural network trained solely on the ERA5 database, as depicted in Fig. 7A,C, shows less distinct shaded areas, highlighting the superiority of the neural network trained on data from the ARPA station in capturing these localized temperature variations.

**Figure 8:**
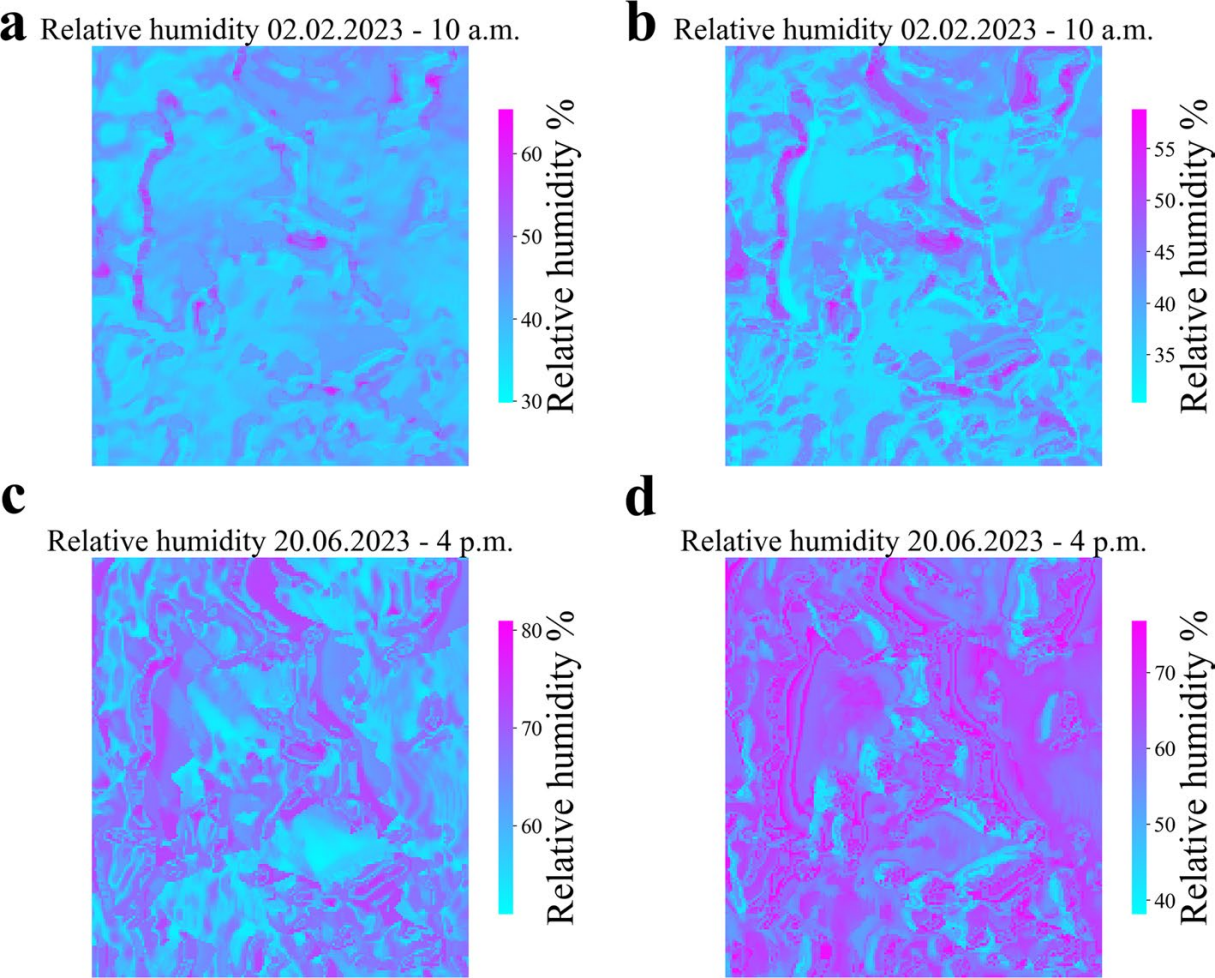
Relative humidity prediction over the entire study area. The figures depict the local relative humidity variation across the entire study area at two specific time points: February 2nd, 2023, at 10 a.m. (**a**,**b**), and June 20th, 2023, at 4 p.m. (**c**,**d**). Figures **a** and **c** show the results obtained from the feed forward neural network trained on the ERA5 database, while figures **b** and **d** present the outcomes from the feed forward neural network trained on the data collected by the ARPA station.

The predictions of relative humidity across the entire study area have also been calculated, and their results for the same time instances mentioned earlier are shown in Fig. 8. However, due to the relatively lower prediction accuracy of the feed forward neural networks trained for relative humidity, the predictions over the entire area may not be entirely reliable. Fig. 8A,B appear to produce reasonably plausible outputs by assigning higher humidity values to the areas covered by shadows. However, in the case of Fig. 8C,D, the predictions do not exhibit a clear and discernible physical pattern.

## Discussion

This paper introduces a sophisticated microclimate model that integrates the principles of physics with deep learning techniques to accurately simulate temperature and humidity variations at a 2-m scale within a study area situated in the Lombardian foothills. The model initially leverages the laws of physics to integrate climate data with terrain properties, downsizing pivotal quantities like radiation and wind to a 2 m resolution. Subsequently, these variables serve as inputs for a feed-forward neural network responsible for predicting temperature and relative humidity variations across the study area. The neural network is trained, validated, and tested using local temperature and relative humidity recordings gathered from a network of 25 sensors distributed throughout the study area.

The model has been tested using two different climate datasets: the global climate dataset ERA5 and data collected from an ARPA meteorological station located nearby the study area. In both cases, the model demonstrates good accuracy in reproducing the local temperature and relative humidity within the study area, especially in predicting temperature. A comparative analysis of the results obtained from the two climate datasets highlights that utilizing local meteorological data (the ARPA station dataset) yields more precise results for microclimate modeling. However, in situations where local data is not available, the use of global climate data (ERA5 dataset) remains a viable and reliable approach. In addition, the use of feed forward neural networks improves the accuracy of temperature and relative humidity prediction respect to the use of a linear regression, given the inherent complex and non-linear nature of microclimate.

To improve the modeling of relative humidity, employing more advanced sensors capable of measuring additional physical variables would be beneficial. Furthermore, refining the comparison between global and local climate data becomes more meaningful when meteorological stations are located within the study area. The results of this study provide valuable insights for selecting appropriate input climate data in microclimate modeling, which has implications for optimizing various essential processes in precision agriculture.

## Methods

### Data

#### Study area terrain data

The framework has been validated in the study area represented in Fig. 1b, located in province of Bergamo, Italy, in the Lombardy foothills (the location of the study area is depicted in Fig. 1a). The center of the study area is located at coordinates $45^\circ$ 23′ 31″ N $9^\circ$ 41′ 48″ E. The terrain morphology has been depicted by a digital surface model which describes the surface altitude at each point with a 2 m resolution, described in Fig. 1c. The study area faces east, with low inclination slopes, and behind it there is a mountain of 700 m high. The study area is surrounded by woods and is currently exploited as mown meadows. In the past, the land has been used for vineyard crops.

The climate of Lombardy, where the study area is located, is mainly humid subtropical (“Cfa” according to the Koppen and Geiger classification^24^), with warm and wet summers and cold winters. However, the Lombardy region exhibits significant climate variations with respect to the Koppen model due to local variability in elevation, proximity to large water basins, and metropolitan areas. Lombardy annual average temperature is around 13.1 °C (55.5 °F), and the average annual rainfall is around 853 mm^25^. The pluviometric regime, in particular, shows two minima in winter and summer and two maxima during spring and autumn seasons.

#### Meteorological data

The meteorological data used as inputs in the microclimate model are sourced from two distinct databases from November 12th, 2022, to June 22nd, 2023, with some pauses in between due to sensors maintenance (from January 15th, 2023, to February 1st, 2023, and from March 7th, 2023, to March 27th, 2023).

The first is the ERA5 database^23^, which generates climate data with a grid resolution of 25 km. In particular the following variables have been considered: total sky direct solar radiation at surface, surface solar radiation downwards, 2 m dewpoint temperature, 2 m temperature, surface pressure, total cloud cover, 10 m U,V wind components and total precipitation. The ERA5 database has been chosen due to its provision of free, high-quality, and reliable data encompassing a wide range of physical variables across global regions.

The second database consists of meteorological data collected by the Lombardy Regional Environment Protection Agency (ARPA) from a meteorological station located 1.3 km away from the study area. As the ARPA station does not measure all the required climate variables for the physical model, the dataset from the ARPA station has been merged with the ERA5 database. Specifically, the ERA5 database provides the radiation parameters, cloud cover, and surface pressure, which are assumed to have minimal variation over the ERA5 grid resolution. On the other hand, the ARPA station measures variables such as temperature, relative humidity, precipitation, wind speed, and wind direction.

These climate data have been combined by the microclimate physical model with the digital surface model to produce the inputs for the feed forward neural networks in order to describe the temperature and relative humidity in each location of the study area.

Figure 2 illustrates the differences in meteorological data between the two databases. As mentioned in the introduction, there are substantial variations in the global climate conditions within a 25 km radius, as recorded by the ERA5 database, compared to the measurements taken at the ARPA station near the study area. These variations form the basis for the current research question, and a meticulous examination of their influence on local microclimate prediction will be undertaken.

It is worth to note that wind direction differences higher than $180^\circ$ have been measured between the two datasets (they have been moduled up to $180^\circ$ by a circular difference in Fig. 2). However, this will not impact the accuracy of the results, as each neural network is trained independently on the ERA5 and ARPA station data, without introducing any relative difference in the model input.

#### Temperature and relative humidity data gathered by monitoring sensors

The temperature and relative humidity data exploited to train, validate and test the feed forward neural networks in the microclimate model have been collected by a network of sensors uniformly deployed over the area, as represented in Fig. 1c.

25 sensors with hygrometers and thermometers have been employed, all of which are based on MEMS technology, to record temperature and humidity levels. These devices boast a temperature measurement range spanning from − 20 to 60 °C, accurate to ± 0.5 °C. In relation to humidity, they can measure values within a 0–99$\%$ range, maintaining an accuracy of ± 5$\%$ RH. The devices transmit data using Bluetooth Low Energy (BLE) connectivity, which ensures a smooth and efficient data collection process. Each sensor has been covered by a wood structure to protect it from the direct contact of precipitation.

The sensors have collected data from November 12th, 2022, to June 22nd, 2023, with some pauses in between due to sensors maintenance (from January 15th, 2023, to February 1st, 2023, and from March 7th, 2023, to March 27th, 2023). Before using the sensors for temperature and humidity measurements in an open field, a calibration process has been carried out under controlled conditions to mitigate potential systematic calibration errors. First, an average temperature and humidity profile has been computed using data from all the sensors. Then, for each individual sensor, the instantaneous deviation from this profile has been calculated and averaged. The resulting values have been established as the calibration parameters, which would subsequently be subtracted from the sensor measurements in the field. By applying this calibration procedure, all systematic calibration errors have been addressed, ensuring more accurate and reliable temperature and humidity measurements from the sensors in the open field. Two different calibration period have been applied: from October 9th, 2022, to October 15th, 2022, and from March 18th , 2023, to March 18th, 2023, to March 23rd, 2023. During both the calibration and recording periods, certain sensors have experienced failures, being unable to capture measurements. The explanation of these failed sensors treatments will be provided in the Feed forward neural networks section.

### Microclimate model

The microclimate model leverages physical laws and feed forward neural networks to obtain a detailed description of temperature and relative humidity at 2 m resolution across the study area. The microclimate model scheme is represented in the supplementary material.

Initially, the meteorological data from the two databases (ERA5 database and ARPA station data) and the terrain data of the study area, represented by the digital surface model, have been given as inputs to the physical model. This model derives meter-scale values of physical variables related to local temperature and relative humidity variations, such as short-wave radiation, long-wave radiation, and wind speed.

Then, these values have been used as inputs to feed forward neural networks to predict the local temperature and relative humidity over specific locations of the study area. The neural network has been trained on temperature and relative humidity data measured by 25 sensors from November 12th, 2022, to June 22nd, 2023, in these specific locations. Part of the sensors data have been used to validate and test the microclimate model accuracy in predicting the local temperature and relative humidity. The trained neural network can be then exploited to predict the temperature and relative humidity over each location of the study area.

In the following, we use the microclimate model proposed by Maclean’s work in 2019^16^ which we implemented in a Python code. In contrast with Maclean’s work, which used a linear regression to model the local variation of temperature, in this paper we apply instead a feed forward neural network, since we expect it will produce better results, given the complexity of microclimate.

#### Physical model

The physical model aims at providing a comprehensive description of the local variations (at a 2 m resolution) of certain physical variables that influence temperature and humidity. It achieves this by using global climate data and leveraging physical laws to establish connections between the local variations of these data and the terrain configuration. For the purpose of this research, the influence of vegetation is not considered. The model focuses on three primary physical variables: short-wave radiation, long-wave radiation, and wind speed. These variables are combined with a feed-forward neural network, which is trained using data collected from sensors, to predict temperature and humidity for each specific location within the study area.

##### Short-wave radiation

Short-wave radiation is the solar radiation received at the ground^6^. It is defined as the sum between the direct and the diffuse solar radiation:

1 $$\begin{aligned} R_{\text{sw}} = (R_{\text{dir}} + R_{\text{dif}}) (1-a) \end{aligned}$$

where the factor *a* is the albedo and describes the amount of reflected radiation. The albedo is set to 0.25 as pointed by^6^.

The direct solar radiation arrives from the direction of the solar disk and includes a small component scattered forward^6^. Its amount on a horizontal surface is obtained from the ERA5 database^23^ as the total sky direct solar radiation at surface (it is measured in $\left[\frac{\text{MJ}}{\text{m}^2 \text{h}}\right]$). Since the terrain conformation is not flat, the radiation has been rescaled as the amount of direct radiation received by an inclined surface, with the slope and aspect of the local terrain (for more details about the slope and aspect please visit the supplementary materials). The flux of radiation that reaches an inclined surface can be computed following^26^ as:

2 $$\begin{aligned} R_{\text{dir}} = \frac{S_{\text{Dir}}}{\cos (\theta )}\zeta [\cos (\theta )\cos (\beta ) + \sin (\theta )\sin (\beta )\cos (\phi -\alpha )] \end{aligned}$$

where $S_{\text{Dir}}$ is the the total sky direct solar radiation at surface obtained from the ERA5 database, $\zeta$ is defined as a binary mask which indicates if the surface is shaded or not, $\theta$ is the sun zenith, $\phi$ is the sun azimuth, $\beta$ is the surface slope and $\alpha$ is the surface aspect (for more details about all these variables please visit the supplementary materials).

The diffuse radiation describes all scattered radiation received from the blue sky and from clouds, either by reflection or transmission^6^. The diffuse radiation on a horizontal surface is computed as the surface solar radiation downwards (the amount of solar radiation that reaches a horizontal plane at the surface of the Earth) minus the direct solar radiation computed as described above (it is measured in $\left[\frac{\text{MJ}}{\text{m}^2 \text{h}}\right]$), obtained from^23^. The diffuse radiation on an inclined surface can be computed as proposed by^27^ dividing it in an isotropic component $R_{\text{I}}$, an anisotropic component $R_{\text{A}}$ and a component reflected by the surroundings $R_{\text{R}}$:

3 $$\begin{aligned} R_{\text{dif}} = R_{\text{I}}+R_{\text{A}}+R_{\text{R}} \end{aligned}$$

The isotropic component $R_{\text{I}}$ can be computed as:

4 $$\begin{aligned} R_{\text{I}} = \frac{1}{2}D(1+\cos (\alpha ))(1-k)s_{\text{vf}} \end{aligned}$$

where *D* is the diffuse radiation on an horizontal surface obtained from^23^, $\alpha$ is the slope of the surface, *k* is the anisotropic index defined as $k = \frac{S_{\text{dir}}}{\cos (\theta ) R_0}$, where $R_0 = 4.87 \frac{\text{MJ}}{\text{m}^2\text{h}}$ and $s_{\text{vf}}$ is the sky view factor (for more details about all these variables please visit the supplementary materials).

The anisotropic component $R_{\text{A}}$ can be computed as:

5 $$\begin{aligned} R_{\text{A}} = \frac{R_{\text{dir}}D}{R_0} \end{aligned}$$

where $R_{\text{dir}}$ is the direct short-wave radiation computed in the section above. $R_{\text{A}}$ describes the circumsolar radiation.

The component reflected by the surroundings $R_{\text{R}}$ can be computed as:

6 $$\begin{aligned} R_{\text{R}} = \frac{1}{2}D a_{\text{r}}(1 - \cos (\alpha + h_{\text{a}})) \end{aligned}$$

where $a_{\text{r}}$ is the mean albedo of the surroundings (it is set to 0.25 as pointed by^6^) and $h_{\text{a}}$ is the mean horizon angle (for more details about all these variables please visit the supplementary materials).

##### Long-wave radiation

Long-wave radiation is the radiation exchanged between the ground and the atmosphere^6^. It can be described treating surfaces as full radiators. A surface with temperature $T_s$ emits radiation as described by (assuming surface emissivity equals to 1):

7 $$\begin{aligned} R_{\text{lwE}} = \sigma T_\text{s}^4 \end{aligned}$$

However it also received some long-wave radiation from the atmosphere as:

8 $$\begin{aligned} R_{\text{lwR}} = \epsilon _m\sigma T_{\text{air}}^4 svf \end{aligned}$$

where $\epsilon _m$ is the emissivity, $\sigma$ is the Boltzmann constant ($\sigma = 5.6693$ 10$^{-8}$Wm$^{-2}$K$^{-4}$) and $s_{\text{vf}}$ is the sky view factor (it takes into account the portion of available sky; it has been described in previous sections). Treating the total long-wave radiation $R_{\text{lw}}$ as outcoming, it can be computed as:

9 $$\begin{aligned} R_{\text{lw}} = R_{\text{lwE}}-R_{\text{lwR}} = (1-\epsilon _{\text{m}} s_{\text{vf}})\sigma T^4 \end{aligned}$$

where small differences between the air and surface temperature have been assumed . The emissivity can be computed from^28^ as:

10 $$\begin{aligned} \epsilon _{\text{m}} = \epsilon _{\text{cs}}F(n) \end{aligned}$$

where $\epsilon _{\text{cs}}$ is the is full-spectrum, clear-sky emittance and *F*(*n*) is the “cloud factor” describing the increase in radiation due to clouds.

$\epsilon _{\text{cs}}$ can be defined from^26^ as:

11 $$\begin{aligned} \epsilon _{\text{cs}} = 1.24(\frac{P_{\text{w}}}{T})^{\frac{1}{7}} \end{aligned}$$

where $P_{w}$ is the water vapor pressure and 1.24 reflects a parameter relationship between vapor pressure and temperature near the ground^29^ [with units of measure of $(\frac{K}{mbar})^{\frac{1}{7}}$]. While *F*(*n*) is defined in^28^ as a weighted sum factor to weight the contribution of the cloud fraction:

12 $$\begin{aligned} F_n = (1-n)+\frac{\epsilon _{\text{oc}}}{\epsilon _{\text{cs}}}n \end{aligned}$$

where $\epsilon _{\text{oc}}$ is the emittance for a totally overcast sky (here set to 1).

It is noteworthy that longwave radiation data can also be obtained from the ERA5 database. However, in the present study, the decision has been made to employ the empirical formulas described above, as done by the work of^16^. Furthermore, the choice of the method for accounting for longwave radiation modelling at 2 m scale should not exert a significant influence on the results of the neural networks since it does not seem to play a crucial role in microclimate modeling, as suggested by Fig. 6.

##### Wind speed

The wind speed at 1 m height, modified by the local terrain conformation, $u_{1m}^*$ has been computed following^16,30^ as:

13 $$\begin{aligned} u_{\text{1m}}^* = u_{\text{1m}}w_{\text{s}} \end{aligned}$$

where $u_{\text{1m}}$ is the wind speed at 1 m (without been modified by the local terrain attributes) and $w_{\text{s}}$ is the topographic shelter coefficient which takes into account how the surface modifies the wind speed (for more details please visit the supplementary materials).

#### Feed forward neural networks

Feed-forward neural networks are considered as the standard architectures for artificial neural networks^31^. Essentially, they can be viewed as functions that take an input and generate an output through hidden mechanisms. The fundamental components of these networks are neurons, which receive input vectors (denoted as $\vec {x}$) and produce outputs ($y_{\text{n}}$) according to the following equation:

14 $$\begin{aligned} y_{\text{n}} = f(\vec {w}^T\vec {x}+b, \theta ) \end{aligned}$$

In this equation, $\vec {w}$ represents a set of weights, *b* is the bias, and *f* is a non-linear activation function. The activation function may also depend on a parameter $\theta$.

Feed-forward neural networks are organized into layers consisting of three types of neuron layers: input, hidden, and output. Neurons within these layers are interconnected, allowing the flow of signals from the input layer to the output layer in a forward direction. The ability of feed-forward neural networks to model input-output relationships relies on the values assigned to the weights of the neurons. These weights are determined using an optimization technique based on the back-propagation algorithm^32^ and gradient descent method^33^.

In the present research, feed forward neural networks trained on the data collected by the sensors have been exploited to predict the temperature and the relative humidity in each location of the study area, starting from the local values of short-wave radiation, long-wave radiation, wind speed, reference temperature, reference relative humidity, precipitation, pressure, cloud cover, specific humidity. The temperature and humidity data collected by the sensors cover the period from November 12th, 2022, to June 22nd, 2023, with some pauses in between due to sensors maintenance (from January 15th, 2023, to February 1st, 2023, and from March 7th, 2023, to March 27th, 2023). The data at first have been aggregated hourly for each sensors and divided in the test, validation and train sets. For the test set, four strategically positioned sensors have been selected based on their uninterrupted recording history without any failures. Another set of four sensors, also chosen strategically, have been designated for the validation set. However, two of these sensors have experienced failures between February 1st, 2023, and March 6th, 2023, one had failures from March 27th, 2023, to June 22nd, 2023, while the remaining sensor had no failures. It is important to note that all the recordings, during a failed period, have been excluded from the validation set for the corresponding sensor. During a scheduled maintenance period (from March 7th, 2023, to March 27th, 2023), the failed sensors have been replaced and calibrated with the non failed sensors. The training phase utilized the remaining 17 sensors. Out of these, nine sensors have encountered some failures and have been handled with the same procedure explained for the validation set. Despite the presence of failures, the uniform distribution of sensors across the study area suggests that neighboring sensors could compensate for the missing information caused by a failed sensor. Therefore, a sensor failure should not significantly hamper the learning process for microclimate prediction.

The feed forward neural networks have been implemented using the powerful Keras and TensorFlow libraries^34^, renowned for their extensive capabilities in deep learning. An example of their architecture is represented in the supplementary material. In the present research, a feed forward neural network with four hidden layers is employed in the case of temperature prediction, with a dimension 1024, 512, 256, and 128 neurons, respectively (both for the prediction on ERA5 and ARPA climate data). While in the case of relative humidity prediction a feed forward neural network is made of 3 hidden layers with 512, 256, and 128 neurons, respectively, when using the ERA5 climate data. While in the case of ARPA meteorological data, the architecture is made of 4 layers with 1024, 512, 256, and 128 neurons. The numbers of hidden layers and neurons have been selected after a hyper-parameters optimization with a grid search process. By incorporating multiple hidden layers, the networks can effectively capture intricate patterns and relationships within the input data, enabling more complex computations and higher-level feature extraction.

To introduce non-linearity and enhance the network’s expressive power, a sigmoidal activation function is chosen for the hidden layers^35^. The sigmoid function effectively maps the neuron outputs into a range between 0 and 1, allowing for the representation of non-linear relationships and ensuring efficient gradient propagation during the training process. The sigmoid function is defined as:

15 $$\begin{aligned} \sigma (x) = \frac{1}{1+e^{-x}} \end{aligned}$$

When evaluating the neural network’s performance, the primary loss function used is the Log-Cosh loss, which is particularly suitable for regression problems^36^. It provides a smooth and robust measure of the discrepancy between predicted and target values, accommodating the potential presence of outliers or extreme values in the dataset. It is defined as:

16 $$\begin{aligned} L(y, y^p) = \sum _{i=1}^N \log (\cosh (y_{\text{i}}^p-y_{\text{i}})) \end{aligned}$$

Furthermore, the mean squared error (MSE) and mean absolute error (MAE) are also calculated as additional evaluation metrics. While they do not directly influence the selection of the best weights during validation, they provide valuable insights into the overall performance and precision of the neural network. MSE measures the average squared difference between predicted and actual values, emphasizing larger errors, while MAE computes the average absolute difference, providing a more intuitive understanding of the average prediction error. They have been defined as follows:

17 $$\begin{aligned} MSE(y, y^p)= & {} \frac{\sum _{i=1}^N (y_{\text{i}}^p-y_{\text{i}})^2}{N} \end{aligned}$$

18 $$\begin{aligned} MAE(y, y^p)= & {} \frac{\sum _{i=1}^N |y_{\text{i}}^p-y_{\text{i}}|}{N} \end{aligned}$$

The Adam optimizer^37^, an advanced gradient descent optimization algorithm, is used to minimize the loss function. Adam stands for “Adaptive Moment Estimation” and is widely recognized for its effectiveness in optimizing neural networks. With Adam, the optimization process is performed by adjusting the network’s weights iteratively, leveraging both the first and second moments of the gradients to adaptively update the learning rate for each weight parameter.

The two feed forward neural networks have been trained for 1000 epochs on the training set to learn the relationship between the local input variables and the local temperature and humidity. During the training phase the value of the loss on the validation set has been tracked and the set of training weights which minimize the validation loss has been chosen. Then the architecture has been tested on the test set and the quality of the prediction has been evaluated through the mean absolute error (MAE) and the determination coefficient $R^2$ (which is the squared Pearson correlation coefficient), where:

19 $$\begin{aligned} R^2(y, y^p) = (\frac{\text{Cov}(y,y^p)}{\sigma _\text{y} \sigma _{\mathrm {y^p}}})^2 \end{aligned}$$

It is important to note that the microclimate prediction is influenced by the daily cycle. Therefore, elevated values of the determination coefficient are expected, especially for temperature predictions. Nevertheless, the determination coefficient can still be a useful metric when comparing the results obtained from the neural networks with the ones obtained with the linear model.

##### Neural networks interpretation

In order to address the inherent black-box nature of neural networks, a method is proposed to interpret the significance of input variables on the output. This approach involves systematically replacing each input variable, one at a time, with random values within its minimum and maximum range while keeping the remaining inputs unchanged. The neural network is then evaluated using this randomized test set, and both the $R^2$ (coefficient of determination) and MAE (mean absolute error) are computed. These values are then compared to the corresponding values obtained from the original non-randomized test set (as obtained in the previous section). To account for statistical variations, this randomization process is repeated multiple times. The mean and standard deviation of the differences in $R^2$ and MAE between the randomized and original scenarios are computed to obtain the final values. If a variable exhibits substantial deviations in both $R^2$ and MAE from the original values, it indicates that it plays a crucial role in the prediction process. In summary, by analyzing the variations in $R^2$ and MAE resulting from input randomization, the importance of each variable in the neural network’s prediction can be determined.

### Supplementary Information

Supplementary Information 1.

## Data Availability

The Pyhton code needed to reproduce the results of the paper and the primary data are available at https://github.com/dndg/MicroclimateDLForecast.

## References

[1] Zellweger F, De Frenne P, Lenoir J, Rocchini D, Coomes D (2019). Advances in microclimate ecology arising from remote sensing. Trends Ecol. Evol..

[2] Chen J (1999). Microclimate in forest ecosystem and landscape ecology: Variations in local climate can be used to monitor and compare the effects of different management regimes. Bioscience.

[3] Geiger R, Aron RH, Todhunter P (2009). The Climate Near the Ground.

[4] Scherrer D, Koerner C (2010). Infra-red thermometry of alpine landscapes challenges climatic warming projections. Glob. Change Biol..

[5] Chen J, Franklin JF (1997). Growing-season microclimate variability within an old-growth douglas-fir forest. Climate Res..

[6] Monteith J, Unsworth M (2013). Principles of Environmental Physics: Plants, Animals, and the Atmosphere.

[7] Tanny J (2013). Microclimate and evapotranspiration of crops covered by agricultural screens: A review. Biosys. Eng..

[8] Schultze SR, Campbell MN, Walley S, Pfeiffer K, Wilkins B (2021). Exploration of sub-field microclimates and winter temperatures: Implications for precision agriculture. Int. J. Biometeorol..

[9] Bwambale E, Abagale FK, Anornu GK (2022). Smart irrigation monitoring and control strategies for improving water use efficiency in precision agriculture: A review. Agric. Water Manag..

[10] Iwasaki K (2019). Spatial pattern of windbreak effects on maize growth evaluated by an unmanned aerial vehicle in Hokkaido, northern japan. Agrofor. Syst..

[11] Pangga IB, Hanan J, Chakraborty S (2011). Pathogen dynamics in a crop canopy and their evolution under changing climate. Plant. Pathol..

[12] Escamilla-García A, Soto-Zarazúa GM, Toledano-Ayala M, Rivas-Araiza E, Gastélum-Barrios A (2020). Applications of artificial neural networks in greenhouse technology and overview for smart agriculture development. Appl. Sci..

[13] Farooq MS, Riaz S, Abid A, Umer T, Zikria YB (2020). Role of iot technology in agriculture: A systematic literature review. Electronics.

[14] Akhter R, Sofi SA (2022). Precision agriculture using iot data analytics and machine learning. J. King Saud Univ. Comput. Inform. Sci..

[15] Kearney MR, Porter WP (2017). Nichemapr-an r package for biophysical modelling: The microclimate model. Ecography.

[16] Maclean IM, Mosedale JR, Bennie JJ (2019). Microclima: An r package for modelling meso-and microclimate. Methods Ecol. Evol..

[17] Maclean IM, Klinges DH (2021). Microclimc: A mechanistic model of above, below and within-canopy microclimate. Ecol. Model..

[18] Klinges DH, Duffy JP, Kearney MR, Maclean IM (2022). mcera5: Driving microclimate models with era5 global gridded climate data. Methods Ecol. Evol..

[19] Maclean IM (2020). Predicting future climate at high spatial and temporal resolution. Glob. Change Biol..

[20] Gardner A, Maclean I, Gaston K, Bütikofer L (2021). Forecasting future crop suitability with microclimate data. Agric. Syst..

[21] Kamilaris A, Prenafeta-Boldú FX (2018). Deep learning in agriculture: A survey. Comput. Electron. Agric..

[22] Bartkowiak P (2022). Land surface temperature reconstruction under long-term cloudy-sky conditions at 250 m spatial resolution: case study of vinschgau/venosta valley in the european alps. IEEE J. Sel. Top. Appl. Earth Obs. Remote Sens..

[23] Hersbach, H. *et al.* Era5 hourly data on single levels from 1979 to present. *Copernicus Climate Change Service (C3S) Climate Data Store (CDS)* **10** (2018).

[24] Beck HE (2018). Present and future köppen-geiger climate classification maps at 1-km resolution. Sci. Data.

[25] Zanchi M (2022). A pipeline for monitoring water pollution: The example of heavy metals in Lombardy waters. Heliyon.

[26] Hengl T, Reuter HI (2008). Geomorphometry: concepts, software, applications.

[27] Hay JE, McKAY DC (1985). Estimating solar irradiance on inclined surfaces: A review and assessment of methodologies. Int. J. Solar Energy.

[28] Konzelmann T (1994). Parameterization of global and longwave incoming radiation for the Greenland ice sheet. Global Planet. Change.

[29] Brutsaert W (1975). On a derivable formula for long-wave radiation from clear skies. Water Resour. Res..

[30] Ryan BC (1977). A mathematical model for diagnosis and prediction of surface winds in mountainous terrain. J. Appl. Meteorol. Climatol..

[31] Svozil D, Kvasnicka V, Pospichal J (1997). Introduction to multi-layer feed-forward neural networks. Chemom. Intell. Lab. Syst..

[32] Werbos, P. Beyond regression: “new tools for prediction and analysis in the behavioral sciences. *Ph. D. dissertation, Harvard University* (1974).

[33] Ruder, S. An overview of gradient descent optimization algorithms. arXiv:1609.04747 (2016).

[34] Abadi, M. *et al.* TensorFlow: Large-scale machine learning on heterogeneous systems (2015). Software available from tensorflow.org.

[35] Szandała T (2021). Review and comparison of commonly used activation functions for deep neural networks. Bio Inspir. Neurocomput..

[36] Saleh, R. A., Saleh, A. *et al.* Statistical properties of the log-cosh loss function used in machine learning. arXiv:2208.04564 (2022).

[37] Kingma, D. P. & Ba, J. Adam: A method for stochastic optimization. arXiv:1412.6980 (2014).

[38] Zanchi A (2022). Interplay of holocene surface faulting and climate in the central po plain, italy. Quatern. Res..

