# Supplementary material for: Harnessing deep learning to forecast local microclimate using global climate data
Source: Sci Rep. 2023 Nov 29;13:21062. doi: 10.1038/s41598-023-48028-1 (PMC10687000; doi:10.1038/s41598-023-48028-1)
Supplement: Supplementary file 1 — Supplementary Information 1. [file 41598_2023_48028_MOESM1_ESM.pdf]

Supplementary materials: Harnessing Deep Learning to Forecast Local Microclimate using Global Climate Data

Marco Zanchi[a,b,*], Stefano Zapperi[b,c,d], Caterina A. M. La Porta[a,b,e,f]

[a]*Department of Environmental Science and Policy, University of Milan, via Celoria 10, 20133 Milano, Italy*
[b]*Center for Complexity and Biosystems, University of Milan, Via Celoria 16, 20133 Milano, Italy*
[c] *Department of Physics, University of Milan, Via Celoria 16, 20133 Milano, Italy*
[d]*CNR - Consiglio Nazionale delle Ricerche, Istituto di Chimica della Materia Condensata e di Tecnologie per l'Energia,Via R. Cozzi 53, 20125 Milano, Italy*
[e]*CNR - Consiglio Nazionale delle Ricerche, Istituto di Biofisica, via Celoria 10, 20133 Milano, Italy*
[f]*Innovation For Well-Being And Environment (CRC-I-WE), University of Milan, Via Celoria 10, 20133 Milano, Italy*

[*]Corresponding author: marco.zanchi@unimi.it; Department of Environmental Science and Policy, University of Milan, Via Celoria 10, 20133 Milan, Italy

1. Supplementary figures

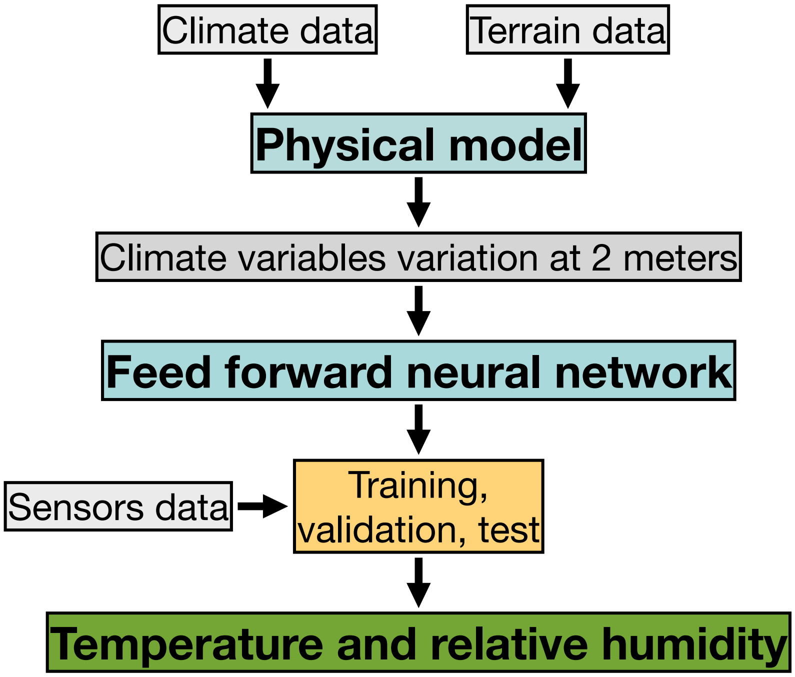

Figure 1: **Microclimate model scheme** The figure illustrates the microclimate model scheme. The model takes as input climate and terrain data and processes them leveraging physical laws to obtain the variation a 2 meters resolution of climate variables across the study area. Then, these variables are given as inputs to a feed forward neural network to predict the local temperature and relative humidity. The neural network is trained, validated and tested exploiting temperature and relative humidity data collected by a network of 25 sensors placed over the study area.

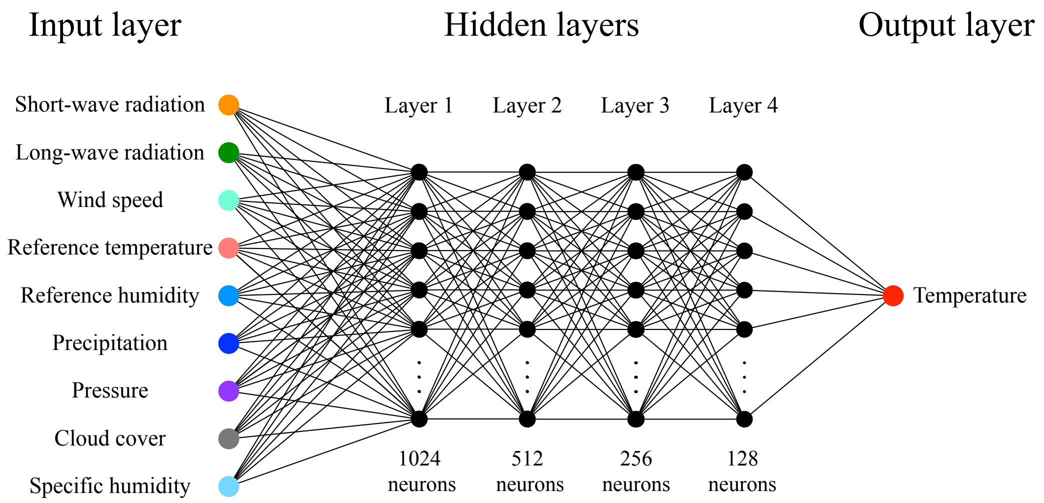

Figure 2: **FFNN architecture.** The figure describes the architecture of the feed forward neural networks exploited for the temperature prediction. It is composed by an input layer with 9 units (short-wave radiation, long-wave radiation, wind speed, reference temperature, reference relative humidity, precipitation, pressure, cloud cover, specific humidity), 4 hidden layers described below and an output layer composed by a dense layer with a single neuron with a linear activation function (it represents the output value of temperature or humidity). The 4 hidden layers are dense layers with a sigmoidal activation function with respectively 1024, 512, 256 and 128 neurons each.

2. Physical model

The microclimate physical model presented here takes inspiration from (1) and it is written in Python. The aim of this model is to describe the local variation (the variation at 2 m resolution) of some physical variables which influence the temperature and humidity variation starting from global climate data. The idea is to exploit physical laws to link the local variation of these global climate data according to the terrain conformation (the effect of vegetation is not taken into account in the present research). The local physical variable described by the model are the shortwave radiation, the longwave radiation, the wind speed. These variables are then combined with feed forward neural network, trained on the data collected by the sensors, to predict the temperature and the humidity in each location of the study area.

2.1. Inputs

The physical model requires different inputs:

- **Time** related inputs are the localtime hour, day, month, year.
- **Space** related inputs are the longitude and latitude of the location of interest.
- **Terrain** related inputs are the digital surface model with a certain resolution of the location of interest.
- **Climate** related inputs are the direct solar radiation, diffuse solar radiation,specific humidity, pressure, fractional cloud cover, precipitation, reference temperature, wind speed and direction.

2.1.1. Terrain input data

The terrain input data describe the terrain conformation. The main data is the digital surface model, a matrix with a certain resolution which describes the elevation of the surface at each point. The digital surface model has been smoothed with an average filter of 4 meters lenght in order to dump the contour lines effects.

2.1.2. Climate input data

Direct solar radiation arrives from the direction of the solar disk and includes a small component scattered forward (2). It is computed as the total sky direct solar radiation at surface (it is measured in $[\frac{MJ}{m^2 hour}]$). The total sky direct solar radiation at surface has been obtained from (3) from *Total sky direct solar radiation at surface* variable.

Diffuse radiation describes all other scattered radiation received from the blue sky and from clouds, either by reflection or transmission (2). It is computed as the surface solar radiation downwards (the amount of solar radiation that reaches a horizontal plane at the surface of the Earth) minus the direct solar radiation computed as described above (it is measured in $[\frac{MJ}{m^2 hour}]$). The surface solar radiation downwards has been obtained from (3) from *Surface solar radiation downwards* variable.

Specific humidity is the mass of water vapor per unit mass of moist air (2). It is dimensionless and it can be computed as:

$$h_s = \frac{m_{water}}{m_{airtotal}} = \frac{m_{water}}{m_{airdry} + m_{water}} = \frac{\rho_{water}}{\rho_{airdry} + \rho_{water}}$$

Exploiting the state equation of perfect gas:

$$\rho_{water} R_{water} T = e$$

where R_{water} is the gas constant of water, T the temperature and e is the actual vapour pressure. The same equation can be written for ρ_{airdry}, opportunely modified with pressure $p - e$, where p is the surface atmospheric pressure in Pa. Substituting in the equation for h_s:

$$h_s = \frac{\frac{e}{R_{water}T}}{\frac{p-e}{R_{airdry}T} + \frac{e}{R_{water}T}} = \frac{e\frac{R_{water}}{R_{airdry}}}{p - (1 - \frac{R_{water}}{R_{airdry}})e} = \\ = \frac{0.622e}{p - 0.378e}$$

The actual vapour pressure in Pa can be computed from (4) as:

$$e = 610.8 exp^{\frac{17.27 T_D}{T_D + 237.3}}$$

where T_D is the dew point temperature (the temperature the air needs to be cooled to, at constant pressure, in order to achieve saturation). The dew point temperature has been obtained from the ERA5 database (3) from *2m dewpoint temperature* variable. Since we are interested in the description of microclimate at 1 meter above the surface we have modified the dewpoint temperature according the lapse rate of $6.50°C/km$ defined by the international standard atmosphere model (ISA):

$$Tdew_{1m} = Tdew_{2m} + 6.50°C/1000$$

Relative humidity is the ratio of air actual vapor pressure to the saturation vapor pressure at the same temperature (2)):

$$h_r(T) = \frac{e}{e_s(T)}$$

where e is the actual vapor pressure and $e_s(T)$ is the saturation vapor pressure at temperature T, which can be computed, in Pa, from (4) as:

$$e_s = 610.8exp^{\frac{17.27T}{T+237.3}}$$

The temperature T has been obtained from (3) from *2m temperature* variable.

Pressure is pressure (force per unit area) of the atmosphere at the surface of land (measured in Pa). The pressure has been obtained from (3) from *Surface pressure* variable.

Fractional cloud cover is the proportion of a grid box covered by cloud. Total cloud cover is a single level field calculated from the cloud occurring at different model levels through the atmosphere (cloud fractions vary from 0 to 1). The cloud cover has been obtained from (3) from *Total cloud cover* variable.

Reference temperature is the temperature at 2 meters high obtained from (3) from *2m temperature* variable. Since we are interested in the description of microclimate at 1 meter above the surface we have modified the reference temperature according the lapse rate of $6.50°C/km$ defined by the international standard atmosphere model (ISA):

$$T_{1m} = T_{2m} + 6.50°C/1000$$

Wind speed (W_{speed}), measured in $\frac{m}{s}$, is computed from the u,v component of the wind at 10 meters (W_U, W_V) high as:

$$W_{speed} = \sqrt{W_U^2 + W_V^2}$$

The u component is the eastward component of the wind, at a height of 10 metres above the surface of the Earth. The v component is the northward component of the wind, at a height of 10 metres above the surface of the Earth. U,V component of the wind have been obtained from (3) from *10 metre U, V wind component* variable. Since we are interested in the microclimate at 1 meter above the surface, we have rescaled the wind speed according the altitude following a logarithmic profile model (2):

$$u_{1m} = u_{10m} \frac{log \frac{h_{1m}}{0.023}}{log \frac{h_{10m}}{0.023}}$$

where $h_{1m} = 1$ and $h_{10m} = 10$ and 0.023 is the surface roughness for grass 0.1 meter high ((2).

Wind direction is computed from the u,v component of the wind at 10 meters high as:

$$W_{direction} = (\arctan(\frac{W_U}{W_V}) \frac{180°}{\pi} + 180°)\%360°$$

The wind direction describes the direction from which the wind comes from with the North direction as reference in decimal degrees ° (wind direction from North is equal to 0°, from East is equal to 90° etc). This convention is taken into account in the above equation: the inverse of the tangent of the ratio between the u,v component of the wind describes the direction in which the wind blows. In order to change the direction (from blowing direction to the direction from which the wind comes from), we have inverted the reference system of the u,v components (the mathematical operation id defined by adding 180° to the angle computed from the inverse of the tangent of the u,v components ratio).

2.2. Model sun coordinates

In order to describe how the terrain influences the solar radiation at surface we need to describe the position of the sun in spherical coordinates (zenith, azimuth) given a certain location defined by a longitude and a latitude and given a particular time defined by hour, day, month, year. At first we describe how to compute the Julian day and the solar time which are need to compute the solar zenith and azimuth and then we describe how to compute the solar zenith and azimuth angles.

2.2.1. Julian day

Julian day is obtained from pandas.Timestamp.tojuliandate. It is the number of days from noon January 1, 4713 BC (without considering the fraction of hours).

2.2.2. Solar time

Local solar time (or simply solar time) is the time according to the position of the sun in the sky relative to one specific location on the ground. We can start from the clock time (the hour on your smartphone) and correct it with 2 factors. The first factor it is related to the change of the longitude inside an certain time zone. The other factor it is described by the equation of time which compute clock hour time corrections due to the obliquity of the ecliptic and to the eccentricity of the sun orbi.

$$T_{solar} = T_{clock} + \frac{4(long_{local} - long_{timezone})}{60} + \frac{EOT}{60}$$

where T_{solar} is the solar time, T_{clock} is the clock time, $long_{local}$ is the local longitude, $long_{timezone}$ is the longitude of the time zone meridian and EOT is the correction in minutes from the equation of time. The units of measure are in hours (since the two correction factors are in minutes we have divided them by 60). Now we are going to describe the two factors. The first factor describes the difference in fraction of hour that there are between the location described by the local longitude and the center of the time zone (the location where the T_{clock} is defined). The factor 4 accounts for the fact that the sun takes 1 hour to do 15° which is the extension of the time zone (there are 12 time zone of 15°). The equation of time it is a phenomenological equation defined as:

$$EOT = -7.659sin(M) + 9.863sin(2M + 3.5932)$$

where M can be computed from the julian day as:

$$M = 6.24004077 + 0.01720197(julian - 2451545)$$

2.2.3. Solar elevation angle and solar zenith angle

The zenith is the point in the sky directly above the observer, and the solar zenith angle is the angle between the sun and zenith with the observer. As a result, in the morning and evening, the zenith angle is highest, while at solar noon, it is minimum. The zenith angle is complementary to the sun elevation angle which is the angular distance between the imaginary horizontal plane on which you are standing and the sun in the sky. The solar elevation angle θ_{elev} for a certain latitude lat can be computed as:

$$sin(\theta_{elev}) = sin(\delta)sin(lat) + cos(\delta)cos(lat)cos(\theta_{solar})$$

where δ is the declination angle of the Earth and θ_{solar} is the solar hour angle (the angular distance travelled by the sun after the solar noon). δ can be computed for each day of the year as:

$$\delta = 23.5cos(\frac{360}{365}(n_{days} - 172)\frac{\pi}{180})\frac{\pi}{180}$$

where θ_{solar} can be computed as:

$$\theta_{solar} = 15\frac{\pi}{180}(T_{solar} - 12)$$

where T_{solar} is the solar time and it has been described in the section above. Finally the solar zenith θ_{zenith} can be computed from θ_{elev} as :

$$\theta_{zenith} = 90^\circ - \theta_{elev}$$

2.2.4. Solar azimuth

The azimuth angle is the compass direction from which the sunlight is coming. At solar noon, the sun is always directly south in the northern hemisphere and directly north in the southern hemisphere. It can be computed as:

$$cos(\theta_{azimuth}) = \frac{sin(\delta)cos(lat) - cos(\delta)sin(lat)cos(\theta_{solar})}{cos(\theta_{elev})}$$

where δ is the Earth declination angle, lat is the latitude, θ_{solar} is the solar hour angle and θ_{elev} is the sun elevation angle.

2.3. Model terrain properties

In this section we will describe some terrain attributes which will be exploited to model how the terrain conformation influences the local variation of microclimate. The terrain is described by a digital surface model, which is a matrix with a certain spatial resolution describing the elevation of the surface at each point. The attributes modelled are the slope and the aspect of the terrain, which indicate how each terrain point is displaced respect to a certain coordinate system, the mean slope and the skyviewtopo, which describes how much a point is surrounded by other higher points, and the lapserate, which describes how the temperature varies with altitude.

2.3.1. Slope

Slope gradient reflects the maximal rate of change of elevation values and is defined in (5) as :

$$Slope = arctan(|\nabla \overline{Z}|)$$

where

$$\nabla \overline{Z} = (\frac{\partial z}{\partial x}, \frac{\partial z}{\partial y})$$

where z is the altitude. Since the dtm is a discrete object we have applied a numerical algorithm to compute the partial derivatives over x and y as proposed by (6):

$$\frac{\partial z}{\partial x} \approx \frac{(z_{0+} - z_{0-})}{2\Delta x}$$

$$\frac{\partial z}{\partial y} \approx \frac{(z_{+0} - z_{-0})}{2\Delta y}$$

2.3.2. Aspect

Aspect indicates the flow-line direction. When water (or any other flowing material) moves downhill under the influence of gravitational force, it will follow the direction specified by aspect, as defined in (5). Aspect can also be used to determine whether a cell should be considered sunny or not. For example, in the northern hemisphere, slopes facing south (i.e. with an aspect of 180°) receive more insolation than those facing north (i.e. with an aspect of 0°) (5). Aspect it is measured in degrees, read clockwise from north, ranging from 0 to 360°. The aspect can be computed as proposed by (5):

$$Aspect = -90[1-sign(q)][1-|sign(p)|]+180[1+sign(p)]-\frac{180}{\pi}sign(p)arccos(\frac{-q}{sqrt(q^2+p^2}))$$

where

$$p = \frac{\partial z}{\partial x}$$

$$q = \frac{\partial z}{\partial y}$$

are computed as described in the aspect section.

2.3.3. Horizon angle

The horizon angle is defined as the maximum angle which hide the horizon along a fixed direction. Fixed the direction it is possible to compute it for each point of the dtm as suggested in (5):

$$HorizonAngle = max_{ij}(arctan(\frac{altidute_{i,j}}{distance_{i,j}}))$$

where (i, j) are all the other dtm location which lies in the fixed direction. The mean horizon angle is defined as the horizon angles averaged over all the possible directions (in practice we have averaged over 36 angular sectors of $10°$).

2.3.4. Sky view factor

The sky view factor is a parameter which describes the proportion of the sky available (for example the proportion of sky not covered by mountains, vegetation, buildings etc). It will be exploited to scale the isotropic diffuse incoming radiation by atmosphere according to the shading of the surroundings. This factor is computed from the mean slope, described in the previous section, as:

$$skyciewfactor = 0.5cos(2MeanHorizonAngle) + 0.5$$

This choice is reasonable since sky view factor must be 0 when the MeanHorizonAngle is $90°$ and 1 when the MeanHorizonAngle is $0°$ (and increases monotonically from $0°$ to $90°$).

2.3.5. Lapserate

The lapserate is the rate at which an atmospheric variable, normally temperature in Earth's atmosphere, falls with altitude. The moist adiabatic lapse rate Γ_m can be defined as:

$$\Gamma_m = g(1 + \frac{L_v r_v}{QT})(c_{pd} + \frac{0.622 L_v^2 r_v}{QT^2})^{-1}$$

where g is gravitational acceleration (9.8076 m/s), L_v is the latent heat of vaporisation (2501000 J/kg), Q is the gas constant for dry air (287 J $kg^{-1}K^{-1}$), c_{pd} is the specific heat of dry air at constant pressure (1003.5 J $kg^{-1}K^{-1}$), T is the reference temperature and r_v is the mixing ratio of water vapour given by:

$$r_v = \frac{0.622e}{P - e}$$

where e is the actual vapor pressure and P is the surface pressure.

The final correction for the lapserate is computed for each point of the dtm as:

$$T = T_{ref} - \Gamma_m(dtm - h_{ref})$$

where T_{ref} is the reference temperature and h_{ref} is the reference altitude (the altitude at which the reference temperature has been computed). In our case T_{ref} has not a defined reference altitude, so we set h_{ref} as the average altitude of our dtm.

2.4. Model shortwave radiation

Shortwave radiation is the solar radiation received at the ground(2). It is defined as the sum between the direct and the diffuse solar radiation:

$$R_{sw} = (R_{dir} + R_{dif})(1 - a) \tag{1}$$

where the factor a is the albedo and describes the amount of reflected radiation. The albedo is set to 0.25 as pointed by (2). In the next chapters we describe how to compute the direct and the diffuse solar radiation for each point of the digital surface model.

2.4.1. Direct radiation

The direct solar radiation arrives from the direction of the solar disk and includes a small component scattered forward (2). Its amount on a horizontal surface is obtained from the ERA5 database (3) as the total sky

direct solar radiation at surface (it is measured in $[\frac{MJ}{m^2 hour}]$). Since the terrain conformation is not flat, the radiation has been rescaled as the amount of direct radiation received by an inclined surface, with the slope and aspect of the local terrain. The flux of radiation that reaches an inclined surface can be computed following (5) as:

$$R_{dir} = \frac{S_{Dir}}{cos(\theta)} \zeta [cos(\theta)cos(\beta) + sin(\theta)sin(\beta)cos(\phi - \alpha)]$$

where S_{Dir} is the the total sky direct solar radiation at surface obtained from the ERA5 database, ζ is defined as a binary mask which indicates if the surface is shaded or not, θ is the sun zenith, ϕ is the sun azimuth, β is the surface slope and α is the surface aspect. The above equation can be explained by the following: the flux of radiation that reaches an inclined surface is the amount of radiation that pass though the same surface but oriented normally to the beam direction rescaled by the cosine of the angle between the two surface. The term $\frac{S_{Dir}}{cos(\theta)}$ describes the amount of radiation that passes through a surface orthogonal to the beam (S_{Dir} it is the radiation trough an horizontal surface when the sun has a zenith angle θ). To compute the amount of solar radiation reaching a generic surface defined by a slope and an aspect we have to multiply the amount of radiation that passes through a surface orthogonal to the beam by the cosine of the angle between this surface and the generic surface defined as $\cos(\gamma)$. The $\cos(\gamma)$ can be computed by the scalar product on spherical coordinates of the normal surface vectors of between the two surfaces (we suppose to work with unit areas for simplicity):

$$\underline{n}_{sun} = (sin(\theta)cos(\phi), sin(\theta)sin(\phi), cos(\theta))$$

where $\underline{n}_{sun}$ is the normal surface vector for the surface orthogonal to the radiation direction, θ is the sun zenith and ϕ is the sun azimuth

$$\underline{n}_{incl} = (sin(\beta)cos(\alpha), sin(\beta)sin(\alpha), cos(\beta))$$

where $\underline{n}_{incl}$ is the normal surface vector for the inclined surface, β is the surface slope and α is the surface aspect.

So now we can compute the scalar product:

$$\begin{aligned} cos(\gamma) &= \underline{n}_{sun} * \underline{n}_{incl} = \\ &= sin(\theta)cos(\phi)sin(\beta)cos(\alpha) + sin(\theta)sin(\phi)sin(\beta)sin(\alpha) + cos(\theta)cos(\beta) = \\ &= cos(\theta)cos(\beta) + sin(\theta)sin(\beta)(cos(\phi)cos(\alpha) + sin(\phi)sin(\alpha)) = \\ &= cos(\theta)cos(\beta) + sin(\theta)sin(\beta)cos(\phi - \alpha) \end{aligned}$$

The shadow mask ζ is computed for each digital surface model point as:

$$\zeta = \begin{cases} 1 & \text{if } \omega < 90^\circ - \theta \\ 0 & \text{if } \omega > 90^\circ - \theta \end{cases}$$

where ω is the horizon angle of the dtm point in the solar azimuth direction.

2.4.2. Diffuse radiation

The diffuse radiation describes all scattered radiation received from the blue sky and from clouds, either by reflection or transmission (2). The diffuse radiation on a horizontal surface is computed as the surface solar radiation downwards (the amount of solar radiation that reaches a horizontal plane at the surface of the Earth) minus the direct solar radiation computed as described above (it is measured in $[\frac{MJ}{m^2 hour}]$), obtained from (3). The diffuse radiation on an inclined surface can be computed as proposed by (7) dividing it in an isotropic component R_I, an anisotropic component R_A and a component reflected by the surroundings R_R:

$$R_{dif} = R_I + R_A + R_R$$

R_I can be computed as:

$$R_I = \frac{1}{2} D(1 + cos(\alpha))(1 - k)svf$$

where D is the diffuse radiation on an horizontal surface obtained from (3), α is the slope of the surface and k is the anisotropic index defined as $k = \frac{S_{dir}}{cos(\theta)R_0}$, where $R_0 = 4.87\frac{MJ}{m^2h}$. The term $\frac{1}{2}(1 + cos(\alpha))$ defines how much horizon the surface can access (if the slope is 0° this therm is equal to 1, if the slope is 90° the available portion of horizon is the half of the total horizon). The svf term shows how much horizon is available (not covered by obstacles).

R_A can be computed as:

$$R_A = \frac{R_{dir} D}{R_0}$$

where R_{dir} is the direct shortwave radiation computed in the section above. It describes the circumsolar radiation.

R_R can be computed as:

$$R_R = \frac{1}{2} D a_r (1 - cos(\alpha + h_a))$$

where a_r is the mean albedo of the surroundings (it is set to 0.25 as pointed by (2), and h_a is the mean horizon angle. The idea behind this equation is to approximate the reflected radiation from the surroundings as the radiation reflected by an infinite surface with slope equal to the average slope of the surroundings (this can be identified by the mean horizon angle).

2.5. Model longwave radiation

Longwave radiation is the radiation exchanged between the ground and the atmosphere (2). It can be described treating surfaces as full radiators. A surface with temperature T_s emits radiation as described by:

$$R_{lwEmitted} = \sigma T_s^4$$

however it also received some longwave radiation from the atmosphere as:

$$R_{lwReceived} = \epsilon_m \sigma T_{air}^4 svf$$

where ϵ_m is the emissivity, σ is the Boltzmann constant ($\sigma = 5.669310^{-8} W m^{-2} K^{-4}$) and svf is the sky view factor (it takes into account the portion of available sky; it has been described in previous sections). Since we treats the total longwave radiation R_{lw} as outcoming (when we will sum it to the shortwave radiation we put a minus in front of it) we write it as:

$$R_{lw} = R_{lwEmitted} - R_{lwReceived} = (1 - \epsilon_m svf) \sigma T^4$$

where we have assumed small differences between the air and surface temperature (note that the temperature used to compute the R_{lw} is the reference temperature corrected by the lapserate). The emissivity can be computed from (8) as:

$$\epsilon_m = \epsilon_{cs} F(n)$$

where ϵ_{cs} is the is full-spectrum, clear-sky emittance and $F(n)$ is the "cloud factor" describing the increase in radiation due to clouds.

ϵ_{cs} can be defined from (5) as:

$$\epsilon_{cs} = 1.24 (\frac{e}{T})^{\frac{1}{7}}$$

where e is the water vapor pressure and 1.24 reflects a parameter relationship between vapor pressure and temperature near the ground (9). While $F(n)$ is defined in (8) as a weighted sum factor to weight the contribution of the cloud fraction:

$$F_n = (1-n) + \frac{\epsilon_{oc}}{\epsilon_{cs}} n$$

where ϵ_{oc} is the emittance for a totally overcast sky (here set to 1). If we combine all the factors mentioned above we obtain:

$$R_{lwReceived} = ((1-n)\epsilon_{cs} + \epsilon_{oc} n)\sigma T^4$$

which describes the radiation emmitted from the atmosphere and received by the surface as a term related to clear sky conditions (the first) and a term which describes an overcast sky(the second).

2.6. Model Wind

The terrain conformation dumps the wind speed u_{1m} according to a topographic shelter coefficient w_s:

$$wind = u_{1m} w_s$$

The idea is to compute the horizon angle in the wind direction (the point in the dtm where the horizon angle is maximum bust be higher than 1 meter, if not the w_s is zero, the wind is not stopped by the topography). Then w_s is defined as suggested in (10):

$$w_s = 1 - \frac{arctan(0.17 tan(Horizon_{angle})100)}{\frac{\pi}{2}}$$

The idea is that if the horizon angle is higher the location will be more protected from the wind (w_s will become less than 1 and so decreases the wind speed at 1 m u_{1m}. The division by $\frac{\pi}{2}$ it is needed to transform the value of the arctan from $[0, \frac{\pi}{2}]$ to [0,1].

References

[1] I. M. Maclean, J. R. Mosedale, J. J. Bennie, Microclima: An r package for modelling meso-and microclimate, Methods in Ecology and Evolution 10 (2) (2019) 280–290.

[2] J. Monteith, M. Unsworth, Principles of environmental physics: plants, animals, and the atmosphere, Academic Press, 2013.

[3] H. Hersbach, B. Bell, P. Berrisford, G. Biavati, A. Horányi, J. Muñoz Sabater, J. Nicolas, C. Peubey, R. Radu, I. Rozum, et al., Era5 hourly data on single levels from 1979 to present, Copernicus Climate Change Service (C3S) Climate Data Store (CDS) 10 (2018).

[4] D. Bolton, The computation of equivalent potential temperature, Monthly weather review 108 (7) (1980) 1046–1053.

[5] T. Hengl, H. I. Reuter, Geomorphometry: concepts, software, applications, Newnes, 2008.

[6] B. K. Horn, Hill shading and the reflectance map, Proceedings of the IEEE 69 (1) (1981) 14–47.

[7] J. E. Hay, D. C. McKAY, Estimating solar irradiance on inclined surfaces: a review and assessment of methodologies, International Journal of Solar Energy 3 (4-5) (1985) 203–240.

[8] T. Konzelmann, R. S. van de Wal, W. Greuell, R. Bintanja, E. A. Henneken, A. Abe-Ouchi, Parameterization of global and longwave incoming radiation for the greenland ice sheet, Global and Planetary change 9 (1-2) (1994) 143–164.

[9] W. Brutsaert, On a derivable formula for long-wave radiation from clear skies, Water resources research 11 (5) (1975) 742–744.

[10] B. C. Ryan, A mathematical model for diagnosis and prediction of surface winds in mountainous terrain, Journal of Applied Meteorology and Climatology 16 (6) (1977) 571–584.
